# *Ancyronyx clisteri*, a new spider riffle beetle species from Borneo, redescription of *A. sarawacensis* Jäch including a description of the larva and new distribution data for *A. procerus* Jäch using DNA barcodes (Coleoptera, Elmidae)

**DOI:** 10.3897/zookeys.912.47796

**Published:** 2020-02-17

**Authors:** Ján Kodada, Manfred A. Jäch, Hendrik Freitag, Zuzana Čiamporová-Zaťovičová, Katarína Goffová, Dávid Selnekovič, Fedor Čiampor Jr

**Affiliations:** 1 Department of Zoology, Faculty of Natural Science, Comenius University, Mlynská dolina B-1, SK-842 15 Bratislava, Slovakia Comenius University Bratislava Slovakia; 2 Naturhistorisches Museum Wien, Burgring 7, A–1010 Wien, Austria Naturhistorisches Museum Wien Wien Austria; 3 Ateneo de Manila University, Biology Department, School of Science and Engineering, Loyola Heights, Quezon City 1101, Philippines Ateneo de Manila University Quezon City Philippines; 4 Universiti Brunei Darussalam, Environmental and Life Sciences, Faculty of Science, Jalan Tungku Link, Gadong, BE1410, Brunei Universiti Brunei Darussalam Gadong Brunei; 5 Taxon Expeditions, Rembrandtstraat 20, 2311 VW Leiden, Netherlands Taxon Expeditions Leiden Netherlands; 6 Zoology Lab, Plant Science and Biodiversity Centre, Slovak Academy of Sciences, Dúbravská cesta 9, SK-84523, Bratislava, Slovakia Plant Science and Biodiversity Centre, Slovak Academy of Sciences Bratislava Slovakia

**Keywords:** Brunei, COI mtDNA sequences, integrative taxonomy, Malaysia: Sabah and Sarawak, spider water beetle, variability

## Abstract

*Ancyronyx clisteri***sp. nov.** (Coleoptera, Elmidae) a new spider riffle beetle discovered from northern Borneo (Brunei; Sabah and Sarawak, Malaysia) and the larva of *Ancyronyx sarawacensis* Jäch are described. Illustrations of the habitus and diagnostic characters of the new species and the similar and highly variable *A. sarawacensis* are presented. Differences to closely related species, based on DNA barcodes and morphological characters, are discussed. Association of the larva and the imago of *A. sarawacensis*, and the occurrence of *Ancyronyx procerus* Jäch in Peninsular Malaysia and Sabah are confirmed by using COI mtDNA sequences.

## Introduction

The first modern taxonomic review of *Ancyronyx* Erichson was published by Jäch (1994) almost 150 years after the description of the genus. Subsequently, numerous new species and larvae were described from Asia (Jäch 2003, 2004; Freitag and Jäch 2007; Freitag and Balke 2011; Bian et al. 2012; Freitag 2012, 2013; Kodada et al. 2014; Freitag and Kodada 2017a, b). Based on these descriptions, the morphological characteristics of the genus were modified (Freitag 2012, Kodada et al. 2014). A total of 33 described species (including one subspecies) is presently known (Jäch et al. 2016, Freitag and Kodada 2017a).

Six species of *Ancyronyx* were so far recorded from Borneo (Brunei; Sabah and Sarawak, Malaysia), the third largest island of the world. However, only few localities in Brunei, Sabah and Sarawak were sufficiently surveyed so far, and the watercourses of Kalimantan largely remain unexplored. Therefore, it can be expected that the fauna of Borneo is probably much richer than known to date. Only a few species, such as *A. acaroides* Grouvelle, *A. malickyi* Jäch and *A. procerus* Jäch, are considered to be widely distributed in Southeast Asia, however, the published distributional data need confirmation at the molecular level. The distribution of most other species is confined to Sulawesi, Indonesia (12 spp.) and some Philippine islands (11 spp.).

Examination of additional material of *Ancyronyx* from Borneo and a detailed study of *A. sarawacensis* Jäch (including type specimens and freshly collected material) revealed an overlooked new species, which is described below.

The fresh material enabled also the use of DNA barcodes to support species delimitation and to estimate the genetic as well as morphological variability of the two species and to describe the larva of *A. sarawacensis*. Furthermore, we were able to confirm the occurrence of *A. procerus* in Peninsular Malaysia by COI mtDNA comparison.

## Material and methods

The material used for study is deposited in the following collections: **BOR/COL** (Borneensis Coleoptera Collection of the Institute for Tropical Biology and Conservation, Universiti Malaysia Sabah, Kota Kinabalu); **CCB** (Collection of Fedor Čiampor Jr, Bratislava, Slovakia); **CFDS** (Collection of Forest Department Sarawak, Kuching, Malaysia); **CFM** (Collection Hendrik Freitag, Manila, currently deposited at the Ateneo de Manila University, Quezon City, Philippines); **CKB** (Collection of Ján Kodada, Bratislava, Slovakia); **NMW** (Naturhistorisches Museum Wien, Austria); **RMNH** (Naturalis Biodiversity Center, Leiden, Netherlands); **UBDM** (Universiti Brunei Darussalam Museum, Brunei).

Dried specimens were soaked in warm water with several drops of concentrated acetic acid and cleaned. Abdomens with genitalia or genitalia only were exposed to lactic acid for one or two days and temporarily mounted onto microscopic slides. Specimens were examined and measured using a Leica M205C stereomicroscope with fusion optics and diffuse lighting at magnifications up to 160 ×. For measurements an eyepiece graticule (5 mm: 100) or the Leica MC190-HD camera attached to microscope and LAS software were used. The specimens were photographed under a Zeiss Axio-Zoom V-16 stereomicroscope using diffuse LED lighting and a Canon 5D Mark IV camera attached. Dissected genitalia and pregenital segments were studied in a temporary microscope cavity slide covered with a cover glass at magnifications up to 640 × with a Leica DM 1000 microscope. All drawings were made using a Leica drawing device.

Principal component analyses (PCA) was performed separately for male and female specimens using software PAST 3.12 (Hammer et al. 2001) and a variance-covariance matrix with log-transformed variables. PCA plots were subsequently edited in Adobe Illustrator CC.

Metric characters of 108 males and 99 females of *A. sarawacensis* as well as 11 males and 20 females of *A. clisteri* sp. nov. were used for the PCA analyses; all specimens identified by mtDNA characters were included in the dataset measured. Morphometric parameters are provided in tables as range and mean ± standard deviation. The following characters were measured: **BL** (body length without head, length of pronotum and elytra measured along midline); **EL** (elytral length, length measured along suture from level of the most anterior point of elytra to the most posterior tip of elytra) in dorsal view; **EW** (elytral width, maximum width combined); **HW** (head width including eyes); **ID** (interocular distance); **MW** (maximum pronotal width); **PL** (pronotal length along midline).

For scanning electron microscopy, specimens were dehydrated in graded ethanol series and then air dried from absolute ethanol, mounted on a stub, sputter coated with gold and viewed and photographed using a TESCAN microscope.

For the DNA analyses, 32 adults (29 *Ancyronyx* spp.; 3 *Graphelmis* spp.) and one larva were used. The dataset is available on dx.doi.org/10.5883/DS-ELMANC01. DNA was isolated from the whole specimens using DNeasy Blood and Tissue Kit (Qiagen) according to the manufacturer’s protocol. Fragment of the 5’ end of the mitochondrial gene for cytochrome c oxidase subunit I (COI) was amplified with primers LCO1490, HCO2198 (Folmer et al. 1994). Amplification products were purified by alkaline phosphatase (FastAP) and exonuclease and sequenced from both sides in Macrogen Europe Inc. (Amsterdam, Netherlands). Raw sequences were assembled and edited in Sequencher v5.1. The genetic distances were measured using K2P model, maximum likelihood tree and bootstrap support were performed in MEGA software v7 (Kumar et al. 2016). The best-fitted substitution model (GTR+I+G) was selected by jModelTest 2 (Darriba et al. 2012). Voucher specimens numbers and GenBank numbers are found between square brackets in the lists of specimens below.

The general morphological terminology follows Kodada et al. (2016) and Lawrence and Ślipiński (2013).

Descriptions of the adults holotypes and mature larvae of *Ancyronyx sarawacensis* are completed with SEM figures of specimens from the respective type locality. Using the standard clearing procedure in lactic acid and gentle pressing by a tip of an entomological pin on the aedeagus delivered extruded endophallus in several males, however the form was well preserved in a single specimen only. The endophallic structures were examined and described only from this *A. sarawacensis* male.

## Results

### DNA analyses

**Figure 1.**
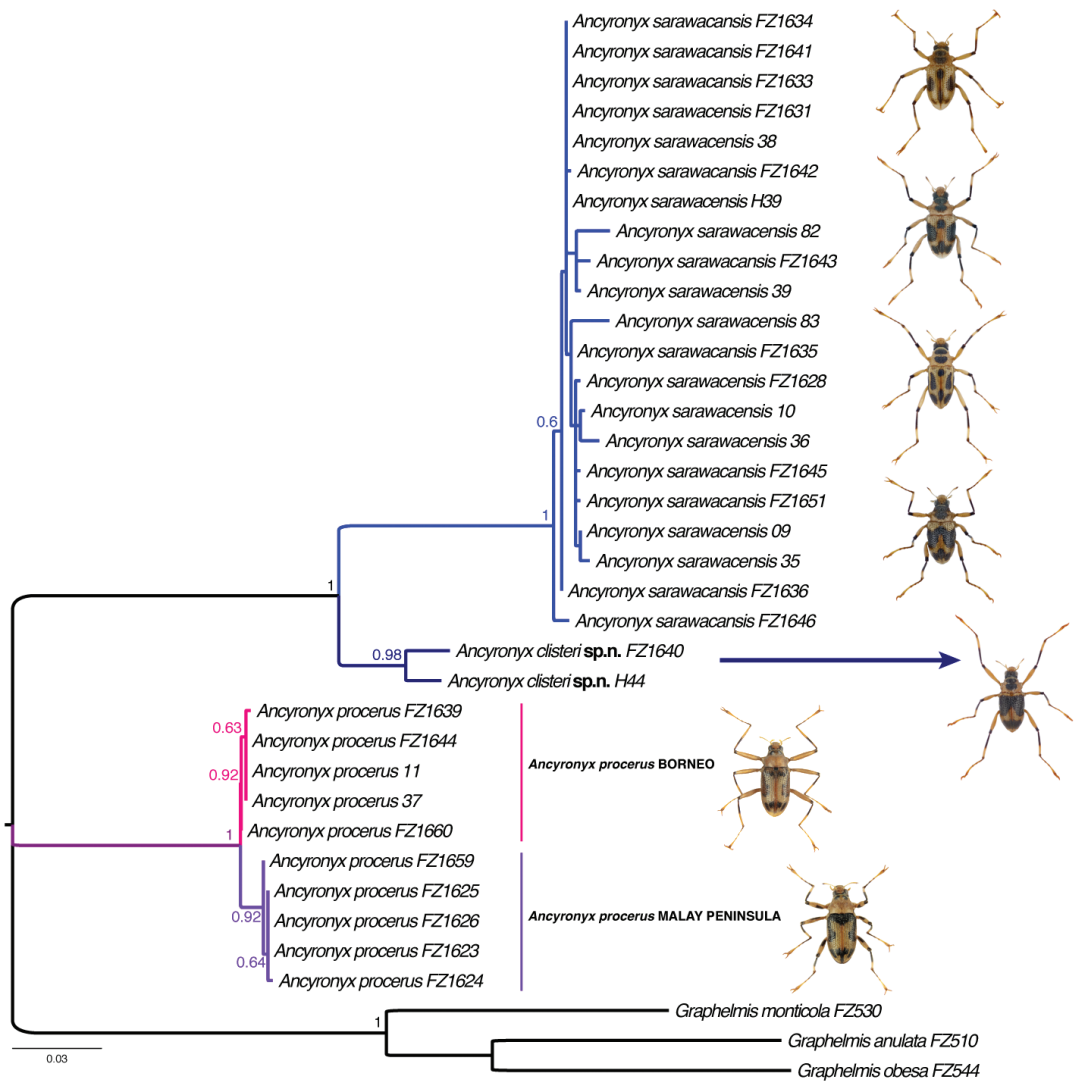
Results of DNA COI analyses, maximum likelihood tree.

The COI sequences used in the analysis are 661bp long with no ambiguous sites or indels. Maximum likelihood (ML) analysis revealed well-separated clades representing three species of *Ancyronyx* (Fig. 1). The interspecific genetic distance was large, ranging from 9.3–17.9%, the intraspecific diversity within *Ancyronyx sarawacensis* ranged from 1.0–3.0%, and within *A. procerus* from 0.0–1.2% (Tab. 2, Suppl. material 1: Tab. S1). In the latter species two genetic lineages were recovered, which correspond with the geographic distribution of the samples. The COI data clearly confirm the occurrence of *A. procerus* in Peninsular Malaysia, which was previously proposed solely based on the morphology of the genitalia.

**Table 1.** Samples used in the molecular analyses: origin of samples, GenBank and BOLD Data Systems BIN accession numbers (codes after species name refer to the voucher numbers used for DNA extraction).

Sample	Country, state	GenBank no.	BOLD BIN no.
*Ancyronyx sarawacensis* FZ1634	Malaysia, Sarawak	**MK505407**	BOLD:ADR1478
*Ancyronyx sarawacensis* FZ1641	Malaysia, Sarawak	**MK505414**	BOLD:ADR1478
*Ancyronyx sarawacensis* FZ1633	Malaysia, Sarawak	**MK505406**	BOLD:ADR1478
*Ancyronyx sarawacensis* FZ1631 / Larva	Malaysia, Sarawak	**MK505395**	BOLD:ADR1478
*Ancyronyx sarawacensis* 38	Malaysia, Sarawak	**MK505398**	BOLD:ADR1478
*Ancyronyx sarawacensis* FZ1642	Malaysia, Sarawak	**MK505418**	BOLD:ADR1478
*Ancyronyx sarawacensis* FRElmH39	Brunei	**LR735552**	BOLD:ADR1478
*Ancyronyx sarawacensis* 82	Malaysia, Sarawak	**MK566773**	BOLD:ADR1478
*Ancyronyx sarawacensis* FZ1643	Malaysia, Sarawak	**MK505415**	BOLD:ADR1478
*Ancyronyx sarawacensis* 39	Malaysia, Sarawak	**MK505401**	BOLD:ADR1478
*Ancyronyx sarawacensis* 83	Malaysia, Sarawak	**MK566771**	BOLD:ADR1478
*Ancyronyx sarawacensis* FZ1635	Malaysia, Sarawak	**MK505422**	BOLD:ADR1478
*Ancyronyx sarawacensis* FZ1628	Malaysia, Sarawak	**MK505420**	BOLD:ADR1478
*Ancyronyx sarawacensis* 10	Malaysia, Sarawak	**MK505409**	BOLD:ADR1478
*Ancyronyx sarawacensis* 36	Malaysia, Sarawak	**MK566772**	BOLD:ADR1478
*Ancyronyx sarawacensis* FZ1645	Malaysia, Sarawak	**MK505404**	BOLD:ADR1478
*Ancyronyx sarawacensis* FZ1651	Malaysia, Sabah	**MK505400**	BOLD:ADR1478
*Ancyronyx sarawacensis* 09	Malaysia, Sarawak	**MK505396**	BOLD:ADR1478
*Ancyronyx sarawacensis* 35	Malaysia, Sarawak	**MK505399**	BOLD:ADR1478
*Ancyronyx sarawacensis* FZ1636	Malaysia, Sarawak	**MK505394**	BOLD:ADR1478
*Ancyronyx sarawacensis* FZ1646	Malaysia, Sarawak	**MK505397**	BOLD:ADR1478
*Ancyronyx clisteri* FZ1640	Malaysia, Sarawak	**MK505421**	BOLD:ADR1475
*Ancyronyx clisteri* FRElmH44	Brunei	**LR735553**	BOLD:AEA6347
*Ancyronyx procerus* FZ1639	Malaysia, Sarawak	**MK505411**	BOLD:ADR0116
*Ancyronyx procerus* FZ1644	Malaysia, Sarawak	**MK505410**	BOLD:ADR0116
*Ancyronyx procerus* 11	Malaysia, Sarawak	**MK505423**	BOLD:ADR0116
*Ancyronyx procerus* 37	Malaysia, Sarawak	**MK505417**	BOLD:ADR0116
*Ancyronyx procerus* FZ1660	Malaysia, Sabah	**MK505403**	BOLD:ADR0116
*Ancyronyx procerus* FZ1659	Malaysia, Pahang	**MK505405**	BOLD:ADR0116
*Ancyronyx procerus* FZ1625	Malaysia, Terengganu	**MK505402**	BOLD:ADR0116
*Ancyronyx procerus* FZ1626	Malaysia, Terengganu	**MK505412**	BOLD:ADR0116
*Ancyronyx procerus* FZ1623	Malaysia, Terengganu	**MK505419**	BOLD:ADR0116
*Ancyronyx procerus* FZ1624	Malaysia, Terengganu	**MK505413**	BOLD:ADR0116
*Graphelmis monticola* FZ530	Malaysia, Kelantan	**MK505416**	BOLD:ADB9822
*Graphelmis anulata* FZ510	Malaysia, Pahang	**MK505424**	BOLD:ADC0259
*Graphelmis obesa* FZ544	Malaysia, Sabah	**MK505408**	BOLD:ADB9823

**Table 2.** Estimates of evolutionary divergence over sequence pairs between groups of three *Ancyronyx* species and the genus *Graphelmis* representing the outgroup.

		**1**	**2**	**3**
**1**	*A. clisteri* sp. nov.			
**2**	*A. procerus*	17.0%		
**3**	*A. sarawacensis*	9.9%	20.1%	
**4**	*Graphelmis* (outgroup)	23.2%	21.2%	23.5%

The two clades recovered in *A. procerus* differ in color patterns, but their genetic differentiation and the subtle differences in their genital morphology are far too weak to consider them as separate species.

### PCA analyses

**Figure 2.**
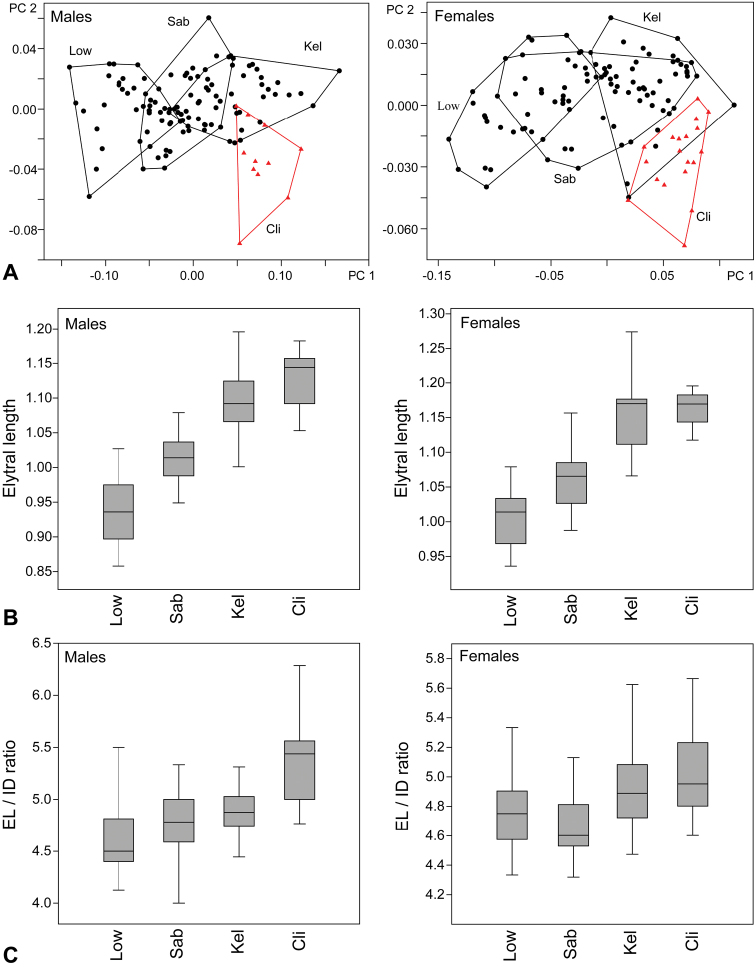
**A** Results of PCA analyses, black circles: specimens of *Ancyronyx sarawacensis*, red triangles: *A. clisteri* sp. nov. Specimens of *A. sarawacensis* are assigned to three groups representing samples from different localities **B** boxplots showing differences in elytral length between *A. clisteri* sp. nov. and three groups of *A. sarawacensis***C** Boxplots showing differences of ratio elytral length / interocular distance between *A. clisteri* sp. nov. and three groups of *A. sarawacensis*. Abbreviations: Low: lowlands of Sarawak, Sab: uplands of Sabah, Kel: Kelabit Highlands, Cli: *A. clisteri* sp. nov.

We examined and quantified the morphometric variations among *Ancyronyx sarawacensis* and *A. clisteri* sp. nov. using PCA (Fig. 2A). First principal component (PC 1) explained 78.18% of variance in males and 76.77% in females. According to loadings (Tab. 3), the strongest correlation was found between body length and elytral length, in both males and females. The second principal component (PC 2) explained 10.15% of variance in males and 9.25% in females and was most strongly correlated with interocular distance, reaching distinctly higher loading compared to other variables in both sexes (Tab. 3).

**Table 3.** The loadings onto the principal components for males and females of *Ancyronyx sarawacensis* and *Ancyronyx clisteri* sp. nov. The first and second highest values for each PC are highlighted in bold.

	**Males**			**Females**		
	**PC 1**	**PC 2**	**PC 3**	**PC 1**	**PC 2**	**PC 3**
Explained Variance (%)	78.18	10.15	4.17	76.77	9.25	6.20
Loadings of Variables:						
BL	**0.450**	-0.310	-0.156	**0.454**	-0.222	-0.300
EL	**0.468**	-0.354	-0.481	**0.448**	-0.143	-0.503
EW	0.418	0.021	-0.073	0.374	**0.147**	-0.223
PL	0.353	-0.105	**0.633**	0.385	-0.473	**0.701**
MW	0.317	0.150	**0.507**	0.298	-0.030	0.168
HW	0.322	**0.169**	0.056	0.312	0.052	0.101
ID	0.274	**0.846**	-0.279	0.345	**0.825**	**0.274**

When assigning the specimens of *Ancyronyx sarawacensis* to three geographically separate groups, i.e., lowlands of Sarawak (40–70 m a.s.l.), uplands of Sabah (170–300 m a.s.l.), and the Kelabit Highlands (around 1000 m a.s.l.), the PCA plot reveals a size gradient related to altitude (Fig. 2A). Specimens from Sarawak lowlands cluster almost separately from those of the Kelabit Highlands. Specimens from Sabah form an intermediate cluster, partly overlapping with both. This gradient corresponds with differences in the actual measurements: elytral length and body length are smallest in the specimens from Sarawak lowlands and largest in the specimens from the Kelabit Highlands (Fig. 2B, Tabs 4, 5).

**Table 4.** Metric characters of *Ancyronyx clisteri* sp. nov. and *Ancyronyx sarawacensis* males. Morphometric parameters are provided as range and mean ± standard deviation.

	*A. clisteri* sp. nov.	*A. sarawacensis*			
	Aggregated data	Kelabit Highland (*N* = 37)	Sabah uplands (*N* = 44)	Sarawak lowlands (*N* = 27)	Aggregated data
**BL: mm**	**1.50–1.64**	**1.38–1.64**	**1.32–1.46**	**1.22–1.40**	**1.22–1.64**
	**1.57** ± **0.04**	**1.49** ± **0.06**	**1.40** ± **0.03**	**1.30** ± **0.05**	**1.41** ± **0.09**
EL: mm	1.05–1.18	1.00–1.20	0.95–1.08	0.86–1.03	0.86–1.20
	1.13 ± 0.04	1.10 ± 0.05	1.01 ± 0.03	0.94 ± 0.05	1.02 ± 0.07
**EW: mm**	**0.70–0.71**	**0.66–0.78**	**0.64–0.72**	**0.59–0.65**	**0.59–0.78**
	**0.71** ± **0.01**	**0.72** ± **0.03**	**0.67** ± **0.02**	**0.62** ± **0.02**	**0.67** ± **0.04**
BL/EW	2.14–2.28	1.88–2.15	1.99–2.20	1.99–2.21	1.88–2.11
	2.21 ± 0.04	2.07 ± 0.06	2.10 ± 0.05	2.10 ± 0.06	2.09 ± 0.05
**EL/EW**	**1.50–1.65**	**1.45–1.64**	**1.42–1.61**	**1.40–1.67**	**1.40–1.67**
	**1.59** ± **0.05**	**1.52** ± **0.04**	**1.51** ± **0.04**	**1.52** ± **0.06**	**1.52** ± **0.05**
PL: mm	0.44–0.48	0.38–0.48	0.38–0.42	0.36–0.40	0.36–0.48
	0.45 ± 0.01	0.41 ± 0.02	0.40 ± 0.01	0.38 ± 0.01	0.40 ± 0.02
**MW: mm**	**0.51–0.57**	**0.49–0.62**	**0.47–0.56**	**0.46–0.55**	**0.46–0.62**
	**0.54** ± **0.01**	**0.54** ± **0.02**	**0.52** ± **0.02**	**0.49** ± **0.02**	**0.52** ± **0.03**
PL/MW	0.79–0.83	0.70–0.85	0.70–0.83	0.70–0.83	0.70–0.85
	0.82 ± 0.02	0.77 ± 0.03	0.77 ± 0.03	0.77 ± 0.03	0.77 ± 0.03
**HW: mm**	**0.39–0.40**	**0.36–0.42**	**0.35–0.40**	**0.33–0.36**	**0.33–0.42**
	**0.39** ± **0.00**	**0.39** ± **0.01**	**0.37** ± **0.01**	**0.35** ± **0.01**	**0.37** ± **0.02**
ID: mm	0.18–0.22	0.21–0.25	0.20–0.25	0.18–0.22	0.18–0.25
	0.21 ± 0.01	0.22 ± 0.01	0.21 ± 0.01	0.21 ± 0.01	0.21 ± 0.01

**Table 5.** Metric characters of *Ancyronyx clisteri* sp. nov. and *Ancyronyx sarawacensis* females. Morphometric parameters are provided as range and mean ± standard deviation.

	*A. clisteri* sp. nov.	*A. sarawacensis*			
	Aggregated data	Kelabit Highland (*N* = 33)	Sabah uplands (*N* = 45)	Sarawak lowlands (*N* = 21)	Aggregated data
**BL: mm**	**1.60–1.70**	**1.44–1.78**	**1.34–1.64**	**1.32–1.50**	**1.32–1.78**
	**1.64** ± **0.03**	**1.59** ± **0.06**	**1.48** ± **0.06**	**1.39** ± **0.05**	**1.50** ± **0.09**
EL: mm	1.12–1.20	1.07–1.27	0.99–1.16	0.94–1.08	0.94–1.27
	1.16 ± 0.02	1.16 ± 0.04	1.06 ± 0.05	1.00 ± 0.04	1.08 ± 0.07
**EW: mm**	**0.70–0.77**	**0.69–0.79**	**0.66–0.78**	**0.62–0.72**	**0.62–0.79**
	**0.73** ± **0.01**	**0.75** ± **0.02**	**0.71** ± **0.02**	**0.65** ± **0.02**	**0.71** ± **0.04**
BL/EW	2.11–2.38	1.94–2.36	1.95–2.23	2.06–2.24	1.94–2.36
	2.25 ± 0.07	2.13 ± 0.07	2.08 ± 0.05	2.14 ± 0.05	2.11 ± 0.06
**EL/EW**	**1.49–1.67**	**1.47–1.62**	**1.36–1.61**	**1.44–1.65**	**1.36–1.65**
	**1.59** ± **0.04**	**1.54** ± **0.04**	**1.50** ± **0.04**	**1.54** ± **0.05**	**1.52** ± **0.05**
PL: mm	0.45–0.52	0.42–0.48	0.39–0.47	0.39–0.44	0.39–0.48
	0.49 ± 0.02	0.44 ± 0.02	0.44 ± 0.02	0.41 ± 0.01	0.43 ± 0.02
**MW: mm**	**0.55–0.61**	**0.53–0.60**	**0.52–0.61**	**0.49–0.55**	**0.49–0.61**
	**0.58** ± **0.02**	**0.56** ± **0.02**	**0.55** ± **0.02**	**0.52** ± **0.02**	**0.55** ± **0.02**
PL/MW	0.80–0.90	0.73–0.86	0.73–0.83	0.74–0.84	0.73–0.86
	0.85 ± 0.03	0.79 ± 0.03	0.79 ± 0.02	0.80 ± 0.03	0.79 ± 0.02
**HW: mm**	**0.39–0.44**	**0.38–0.43**	**0.36–0.44**	**0.35–0.39**	**0.35–0.44**
	**0.41** ± **0.01**	**0.40** ± **0.01**	**0.39** ± **0.02**	**0.37** ± **0.01**	**0.39** ± **0.02**
ID: mm	0.21–0.25	0.21–0.25	0.21–0.25	0.20–0.23	0.20–0.25
	0.23 ± 0.01	0.24 ± 0.01	0.23 ± 0.01	0.21 ± 0.01	0.22 ± 0.01

The cluster of *Ancyronyx clisteri* sp. nov. overlaps with the Kelabit Highlands cluster of *A. sarawacensis* (Fig. 2A, B), but it is clearly separated along the PC 1 axes from clusters of Sarawak lowlands and Sabah uplands. *Ancyronyx clisteri* sp. nov. is also differentiated from *A. sarawacensis* along the PC 2 axes correlating with interocular distance and head width (Fig. 2A) and males of the two species overlap only very marginally in their EL/ID ratios (Fig. 2C).

#### Ancyronyx clisteri sp. nov.

Taxon classificationAnimaliaColeopteraElmidae

DC9FC30C-5442-5FCB-9CF8-C66EA8503258

http://zoobank.org/201B4FB8-C588-468F-892A-A6A8F62FFEAF

##### Type locality

(Fig. 11A). River, about 10 m wide (tributary of Kuamut River near Kampung Pisang Pisang), meandering, with submerged wood; Sabah, Malaysia.

##### Type material.

***Holotype*** ♂ (NMW): “Malaysia, Sabah, Kuamut river env. near Kampung Pisang Pisang, 3.–4. VII. 1996, 14b: ca 10 m wide tributary of Kuamut River in primary forest”. ***Paratypes*** (BOR/COL, CCB, CFDS, CFM, CKB, NMW, RMNH, UBDM): 1 ♂, 2 ♀♀: same locality data as holotype; 5 ♂♂, 8 ♀♀: “Malaysia, Sabah, (Borneo), Kuamut river env. near Kampung Pisang Pisang, 3.–4. VI. 1996, 14a: shaded stream in primary forest with submerged wood”; 2 ♀♀: “Malaysia, Sabah, Kampung Pisang Pisang env., tributary of Kuamut River, 29. VI. 1998”; 4 ♂♂, 7 ♀♀: “Malaysia, Sabah, Sabalangang river in primary forest ca 25 km SE Sapulut, 26.06.1998”; 1 ♀: “Malaysia, Sabah, ca 5 km S Sapulut, Saliku river,16.V.2001”; 1 ♀: “Malaysia: Sabah: Maliau Basin Studies Center: Kuamut River tributary, road bridge near observation tower; submerged wood, riffle; 4°42'48"N, 116°58'34"E, 280 m a.s.l.; 03.Oct2017, leg. H. Freitag & C.V. Pangantihon / Taxon Expeditions (KRC1f)”; 1 ♀ [FZ1640, MK505421]: “Malaysia, Sarawak, Marudi distr., Gunung Mulu NP, 17.10.2018, (42) 04.0267N, 114.818083E, 60 m a.s.l., river, J. Kodada & D. Selnekovič lgt.”; 5 ♂♂ [H44, LR735552], 7 ♀♀: “Brunei: Temburong, Belalong River tributary Sungai Sibut; W of Ashton Trail, submerged wood, run; 4°32'38"N, 115°08'51"E, 170 m a.s.l.; leg. Pangantihon / Taxonexpeditions 29.Sep2018 (SiCf)M”.

##### Diagnosis.

**Figure 3.**
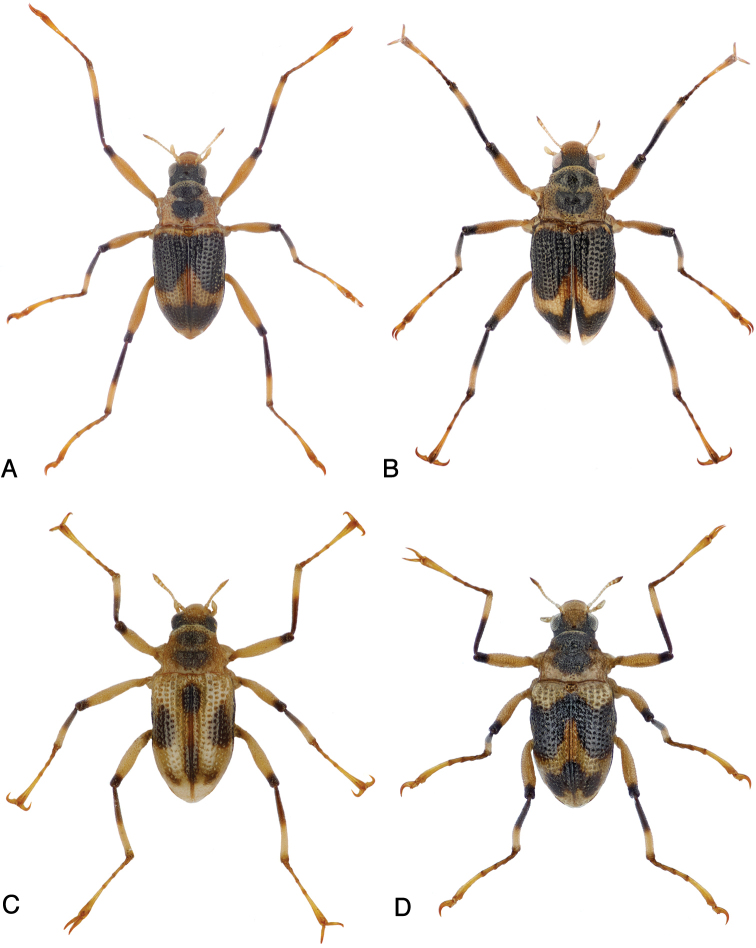
Habitus of: **A***Ancyronyx clisteri* sp. nov., holotype **B***A. clisteri* sp. nov., female paratype from Gunung Mulu NP, Sarawak **C***A. sarawacensis*, holotype **D***A. sarawacensis*, male from a tributary of Kuamut River “(14a)”, Sabah.

**Figure 4.**
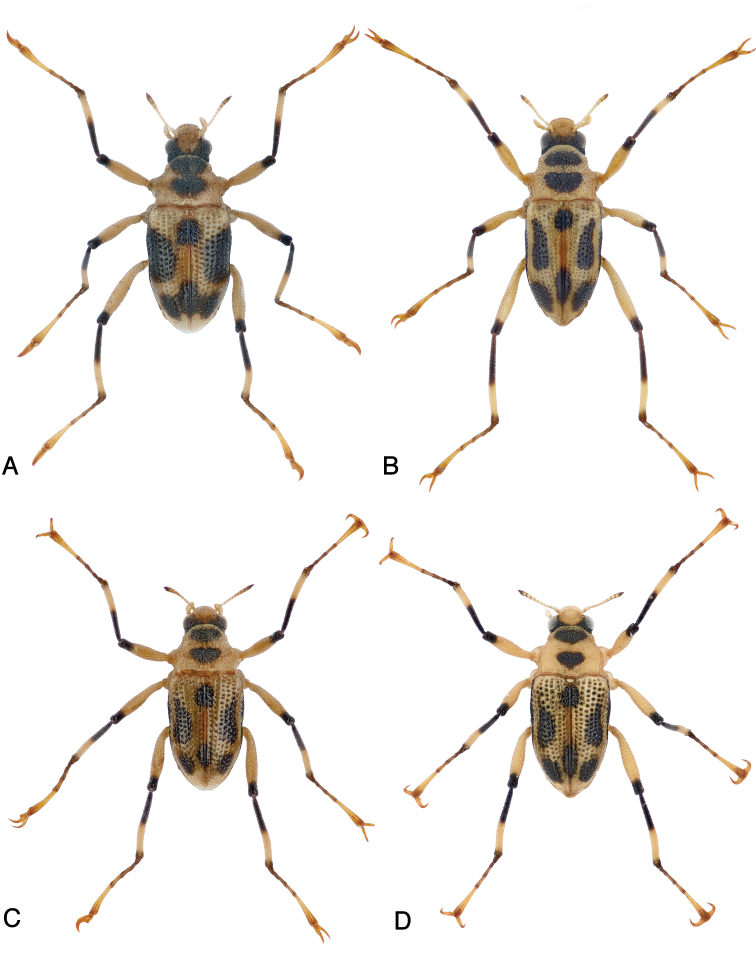
Habitus of: **A***Ancyronyx sarawacensis*, male from the tributary of Kuamut River “(14a)”, Sabah **B***A. sarawacensis*, male paratype from Kapit area, Sarawak **C***A. sarawacensis*, female paratype from Gunung Mulu NP, Sarawak **D***A. sarawacensis*, female from Bayur River near Kampung Bayur, Sarawak.

*Ancyronyx clisteri* sp. nov. is a medium sized, elongate species with dark head and elytra (Fig. 3A, B). It is morphologically most similar to *Ancyronyx sarawacensis* from which it can be distinguished by: 1) elytra extensively black with a very narrow yellowish band along anterior margin and a moderately wider yellowish portion dividing anterior and posterior black area; 2) ovipositor with longer and narrower distal portion of coxite (2.7–2.9 × as long as wide near middle); 3) proximal portion of coxite ca 0.7–0.8 × as long as distal portion; 4) longitudinal baculum of paraprocts ca 1.04–1.19 × as long as entire coxite length; 5) about 9% divergence of the partial mtDNA for cytochrome c oxidase subunit COI (COI barcodes with 90.7% similarity between *A. clisteri* sp. nov. and *A. sarawacensis*, based upon 661 base pairs). We were unable to find significant differences between the aedeagi of these two species, although in direct comparison the apex of the penis of *A. clisteri* sp. nov. usually appears to be narrower and more pointed than that of *A. sarawacensis*. Some *A. sarawacensis* from Sabah (e.g., Figs 3D, 4A) show darker heads and pronota and wide anterior and posterior elytral spots, their color pattern is very similar to those of *A. clisteri* sp. nov. (see respective comment on variability below). For their correct identification it is best to use the COI barcodes or comparison of ovipositors. It is possible that some of the dark males of *A. sarawacensis* cannot be identified by morphological features without direct comparison with *A. clisteri* sp. nov.

##### Description of holotype.

Body form moderately elongate, elytra moderately convex dorsally, with highest point near midlength; BL: 1.64 mm, EW: 0.74 mm, BL/EW: 2.22.

Coloration (Fig. 3A, B). Labrum yellowish-brown; mouth parts and antennae yellowish, clypeus and narrow anterior portion of frons yellowish, remaining portions of cranium black dorsally and posteriad of eyes; pronotum yellowish with large mesal black spot; spot distinctly narrowed near middle; scutellum brownish; elytra mostly black, with yellowish anterior margin and two oblique yellowish stripes meeting at suture; elytral apices yellowish. Venter yellowish except for almost black mesanepisterna, metanepisterna, lateral portion of metaventrite and lateral portions of ventrite 1; coxae yellow; femora black on distal one sixth, yellowish on remaining portion; tibiae black on proximal three fourths and near articulation with tarsi, yellowish on distal fourth; tarsomeres 1–2 darker, tarsomeres 3–5 and claws yellowish.

**Figure 5.**
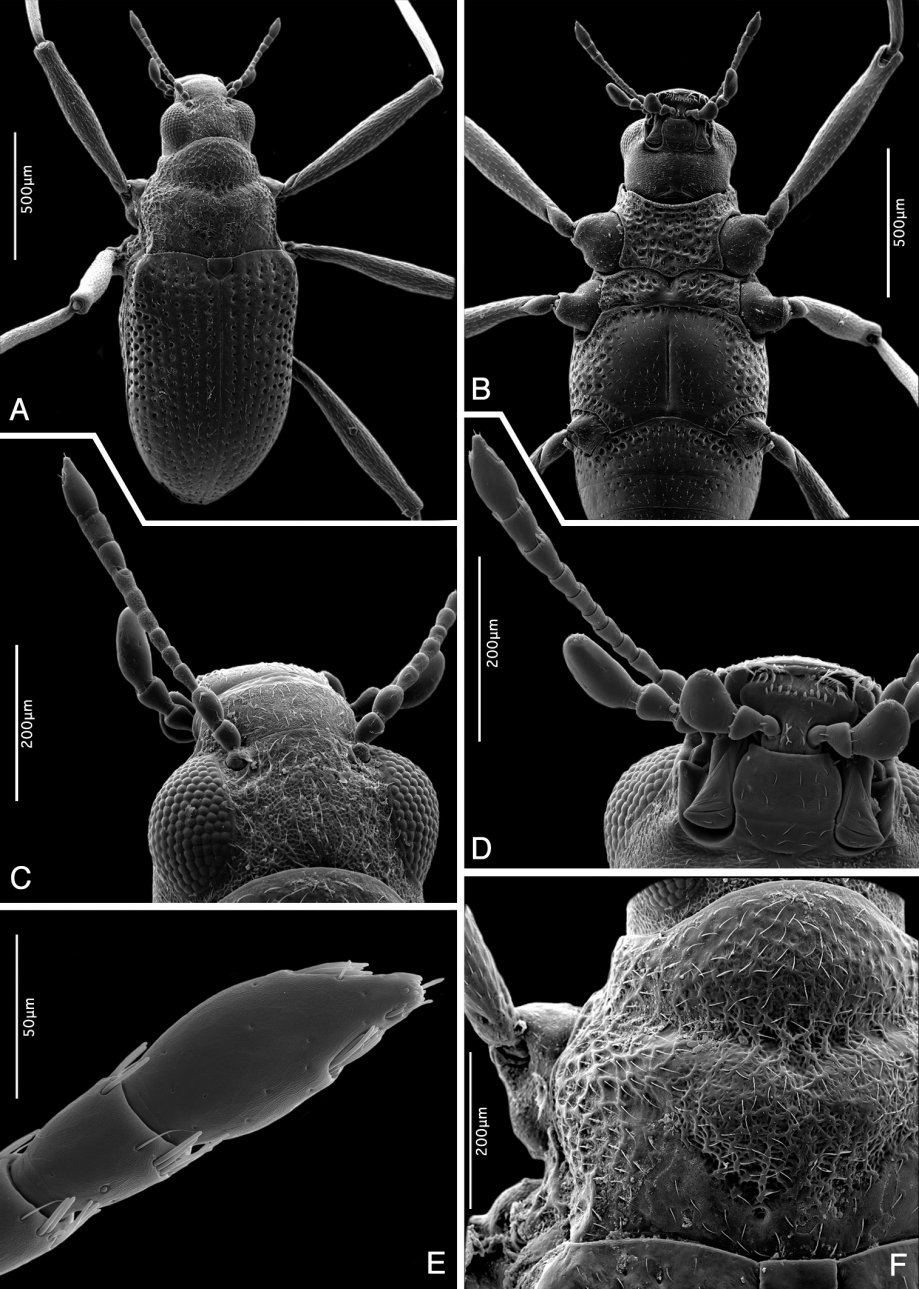
*Ancyronyx clisteri* sp. nov., SEM micrographs, male paratype from the type locality: **A** habitus, dorsal view **B** same specimen, ventral view **C** head, reticulate surface and granules, dorsal view **D** head, detail of mouth parts and antenna, ventral view **E** antennal apex with clusters of sensilla, ventrolateral view **F** pronotum with reticulate surface structure, dorsal view.

Head. Labrum about as long as clypeus, with anterior margin slightly concave, almost straight; surface with dual punctation; larger punctures deeper with fine setae, smaller punctures very fine and shallow. Clypeus wide, densely punctate and finely reticulate. Frons and vertex densely, finely punctate, appearing reticulated; reticulation more distinct on black portion of vertex; surface with narrow, elongate, hardly discernible granules (Fig. 5C); frontoclypeal suture almost straight, finely impressed. Eyes moderately protruding and large. Antennae 11-segmented, subequal in length with pronotum; each antennomere with a few scattered trichoid setae (sensilla trichoidea); antennomeres 9–11 each with two clusters of peg-like setae near distal margins (Fig. 5D, E); terminal segment with additional different sensilla. Ratio of length of antennomeres 1–11: 0.049 : 0.053 : 0.039 : 0.028 : 0.031 : 0.028 : 0.037 : 0.033 : 0.041 : 0.045 : 0.093 mm. Gena microsculptured; gula narrow, smooth; gular sutures absent; posterior tentorial pits deep and large. HW: 0.39 mm; ID: 0.22 mm.

**Figure 6.**
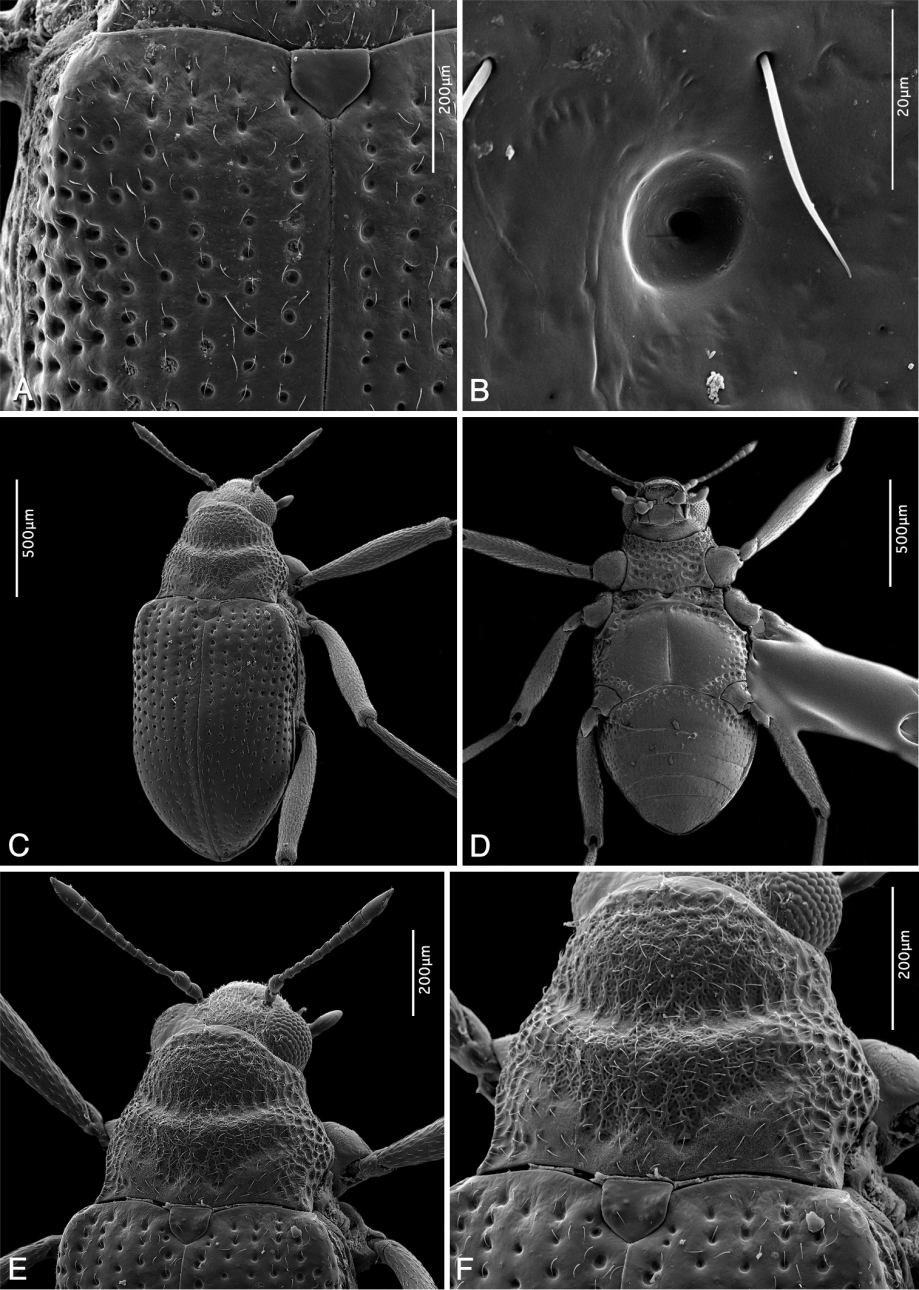
SEM micrographs: **A, B***Ancyronyx clisteri* sp. nov. (male paratype from the type locality) **C–F***A. sarawacensis* (female from the type locality). **A** Elytra and scutellum, anterior portion, dorsal view **B** same, detail of elytral surface, dorsal view **C** habitus, dorsal view **D** same, ventral view **E** head, pronotum and anterior portion of elytra, dorsal view **F** pronotum with reticulate surface structure, dorsal view.

Thorax. Pronotum distinctly wider than long (PL/MW: 0.84), widest near posterior angles; anteriorly attenuate; anterior margin strongly arcuate; almost entire hypomeral portion visible in dorsal view; anterior transverse groove distinctly impressed, oblique and dividing pronotum; area posteriad of transverse groove strongly gibbous; anterior mesal longitudinal carina absent; posterolateral oblique grooves moderately impressed. Pronotal surface densely punctate and irregularly reticulate on disc; anterior and posterior portion smooth (Fig. 5A, F); flat cordiform granules mainly laterally and anteriorly; PL: 0.48 mm, MW: 0.57 mm. Prosternum irregularly, densely and roughly punctate, very short in front of procoxae; prosternal process distinctly transverse, almost flat; posterior margin widely rounded, feebly protruding posteriad; lateral margins arcuate. Scutellum subpentagonal, smooth and shiny. Elytra elongate; EL: 1.18 mm; slightly narrowed at the level of metacoxae, then gradually convergent towards conjointly rounded apices; with ten more or less regular rows of punctures; six rows between suture and shoulder; accessory scutellary rows absent (Fig. 6A); punctures large, round and deeply impressed on disc and laterally (Fig. 6A, B), smaller and less distinct anteriorly and posteriorly; interstices and intervals wider and flat on disc, narrower and feebly convex laterally and posteriorly; surface very finely sculptured; humeri prominent. Mesoventrite almost flat, approximately half as long as prosternum length; mesoventral cavity shallow and narrow; surface strongly and irregularly punctate; mesoventral discrimen invisible; posterior angles rounded and moderately protruding. Metaventrite along midline distinctly longer than combined length of prosternum and mesoventrite; anterior margin arcuate; disc with shallow longitudinal depression mesally; discrimen strongly depressed (Fig. 5B); surface of disc glabrous with fine scarce punctures; distinct, deep irregular punctures along anterior margin and on lateral portions of metaventrite; punctures coarser and denser laterally. Hind wings fully developed. Forelegs about 1.47 × as long as body length; pro- and mesocoxae large and prominent, strongly protruding laterad, bluntly drop-shaped; metacoxae smaller and less protruding laterad; femora, tibiae, and tarsi with short setae; tibiae with a few additional longer setae; distal tarsal segments with several longer setae near apex; claws large, strongly curved, base with two small teeth.

Abdomen. Abdominal intercoxal process moderately longer than length of ventrite 1 posterior of metacoxae, very wide, anteriorly widely arcuate, with rows of deep large punctures along anterior margin; ventrite 1 longest; ventrite 2 ca 0.75 × as long as ventrite 1; ventrite 3 ca 0.88 × as long as ventrite 2; ventrite 4 ca 0.75 × as long as ventrite 3; ventrite 5 as long as combined lengths of ventrites 3 and 4. Surface of ventrites 2–5 with sparse punctures and setigerous flat, more or less cordiform granules; punctures more distinct on mesal portion; granules more prominent and more conspicuous laterally; ventrite 5 granulate. Male sternite IX ca 340 μm long; apical margin arcuately, but distinctly emarginated; lateroapical portion with a few moderately long setae; paraprocts not reaching beyond apical margin. Tergite VIII finely reticulate, with conspicuous median transverse ridge separating posterior and anterior portion; basal half with microtrichial pattern; apical margin hyaline, with subapical fringe of hair-like setae; setae on sublateral portions stronger and longer than those along margin.

Aedeagus (Fig. 13A, B) ca 350 μm long; penis (including lateral basal apophyses) ca 2.85 × as long as phallobase, gradually tapering apicad; apical half moderately curved ventrad (LV), dorsolateral portion with only a few very short setae; apex narrowly rounded; lateral basal apophyses long; ventral sac large; fibula conspicuous, moderately wide; surface of endophallus with spinules; corona indistinct. Phallobase asymmetrical; parameres moderately exceeding middle of penis, widest near base, narrowed to apex; dorsal margin arcuate; ventral margin feebly sinuate; apex narrowed and rounded; mesal and outer surface of parameres with short setae.

##### Description of ovipositor

(Fig. 14A–C). Ovipositor ca 490 μm long; stylus narrow and long, almost straight, ca 0.50 × as long as coxite (compared to the length of distal portion of coxite). Coxite moderately long and stout, rounded at posterolateral angle; distal portion ca 2.70–2.90 × as long as wide near middle, slightly bent, with numerous short stout peg-like setae and with a few thinner peg-like setae; latter mainly at apical portion; inner margin moderately pubescent; proximal portion ca 0.70–0.80 × as long as distal portion, with peg-like and short, fine hair-like setae. Transverse baculum well sclerotised; longitudinal baculum of paraprocts (valvifers) ca 1.04–1.19 × as long as coxite (measured from apical margin of coxite to point where it is joining the transverse baculum).

##### Secondary sexual dimorphism.

Not strongly pronounced. Females on average longer and wider than respective males, with longitudinal depression of metaventrite narrower and shallower. Ventrite 5 in females longer and narrower than in males.

##### Variability.

The specimens vary moderately in size (Tabs 4, 5). The elytral coloration exhibits, in contrast to *Ancyronyx sarawacensis*, only minor variation: some specimens have a larger yellowish anterior margin, oblique yellowish stripes and yellowish apices. In the genitalia, we detected only minor variability in the form of the penis. However, the dataset is too limited for a well supported statistical analysis.

##### Habitat.

The type locality is a shallow meandering river of about 10 m width flowing through primary forest, with stony substrate and plenty of submerged wood (Fig. 11A). Specimens were collected exclusively from submerged wood predominatly in stream reaches with relatively strong current. The specimens from Brunei were collected in a very similar habitat – an upland headwater stream in (slightly disturbed) primary forest (Fig. 12A); the small piece of submerged wood from which the specimens were collected was found in a very shallow, slowly flowing reach (Fig. 12A). A single female was collected from submerged wood in fast flowing, shallow water in a small tributary in primary rainforest (280 m a.s.l.) near the Maliau Basin, Sabah. The altitude of all collection sites ranges from 60–300 m a.s.l. The limited number of collection sites suggests that this species is restricted to rather pristine forest streams at lower altitudes.

##### Syntopic taxa.

At the type locality specimens were collected together with *Ancyronyx acaroides*, *A. procerus* and *A. sarawacensis*, all found on the same submerged tree trunk. Some species of *Graphelmis* Delève (Elmidae), *Elmomorphus* Sharp and *Stenomystax* Kodada, Jäch & Čiampor (Dryopidae) also occurred in the same microhabitat. However, in Temburong, besides *Ancyronyx acaroides*, and *A. sarawacensis*, no other species were collected from exactly the same piece of submerged wood.

##### Distribution.

This species is presently known from a few localities in northern Borneo: Brunei, Sabah and Sarawak.

##### Etymology.

The species is named after Clister V. Pangantihon of the Ateneo de Manila University (Philippines), assistant at Taxon Expeditions, who discovered the new species during the expedition to Brunei. The name was selected by Taxon Expedition participants, instructors and staff of the Kuala Belalong Field Studies Centre in recognition for Clister’s discovery and his most appreciated engagement and friendly care for the expedition participants.

#### Ancyronyx sarawacensis

Taxon classificationAnimaliaColeopteraElmidae

Jäch, 1994

11E8E89F-33BD-5E4B-BE6E-66E2C0FD7237

##### Type locality

(Fig. 11C). Small stream, ca 3 m wide, flowing through degraded primary forest above the village of Arur Dalan near Bario, ca 1000 m a.s.l., Kelabit Highlands, northern Sarawak, Borneo, Malaysia.

##### Material examined. Adults.

***Holotype*** ♂ (NMW): “Malaysia, Sarawak 1993 Kelabit HL. Umg. Bario, 26.2., ca 1000 m leg. M. Jäch (14)”. ***Paratypes*** (NMW, CKB): 1 ♀, 2 unsexed exs: “Malaysia, Sarawak 1993 Kelabit HL., 5 km E Bario Pa Ukat, 27.2., 1000 m leg. M. Jäch (15)”; 1 ♂, 1 ♀: “MALAYSIA, Sarawak 1993 Kelabit HL., 5 km E Bario Pa Ukat, 1.3., 1000 m leg. M. Jäch (17)”; 1 ♂, 5 unsexed exs: “Malaysia, Sarawak Mulu NP, Long Iman 4.3.1993 leg. M. Jäch (20)”; 4 ♂♂, 2 ♀♀, 8 unsexed exs: “Malaysia – Sarawak 40 km E Kapit III. 1994 leg J. Kodada / Rumah Ugap Ng marating Kapit Sut”.

##### Additional material

(BOR/COL, CCB, CFDS, CFM, CKB, NMW, UBDM, RMNH). **SARAWAK**: 1 ♂: “Malaysia, Sarawak, Marudi distr., Gunung Mulu NP, 16.10.2018, (40) 04.0267N, 114.8234E, 55 m a.s.l., small stream, J. Kodada & D. Selnekovič lgt.”; 1 ♂ [FZ1634, MK505407], 1 ♀ [38, MK505398], 1 ♂ [39, MK505401]: “Malaysia, Sarawak, Miri distr., Bario env., 20.06./24.06.2018, (7) 03.76665N, 115.45371E 1146 m a.s.l., Arur Takang, J. Kodada & D. Selnekovič lgt.”; 1 ♂: “Malaysia, Sarawak, Miri distr., Bario env., 20.06.2018, (8) 03.76567N, 115.45215E 1121 m a.s.l., Arur Takang, J. Kodada & D. Selnekovič lgt.”; 1 ♂ [FZ1641, MK505414], 1 ♀: “Malaysia, Sarawak, Miri distr., Ramudu env., 27.06.2018, (16) 03.56813N, 115.49488E 919 m a.s.l., Pa’Ngaruren riv., J. Kodada & D. Selnekovič lgt.”; 1 ♂ [FZ1633, MK505406], 2 ♀♀: “Malaysia, Sarawak, Miri distr., Bario env., 19.06.2018, (6) 03.74350N, 115.43137E 1131 m a.s.l., Pa’Ramapoh, J. Kodada & D. Selnekovič lgt.”; 1 ♂ [82, MK566773], 1 ♂ [83, MK566771], 9 ♂♂, 9 ♀♀: “Malaysia, Sarawak, Miri distr., Bario env., 5.10.2018, (33) 03.759617N, 115.440233E 1143 m a.s.l., Arur Dalan, J. Kodada & D. Selnekovič lgt.; 1 ♂, 1 ♀: “Malaysia, Sarawak, Miri distr., Bario env., 22.06.2018, (10) 03.76245N, 115.43778E 1167 m a.s.l., Arur Dalan, J. Kodada & D. Selnekovič lgt.”; 7 ♂♂, 7 ♀♀: “Malaysia, Sarawak, Miri distr., Bario env., 21.06.2018, (9) 03.76894N, 115.44592E 1171 m a.s.l., Pa’Marario, J. Kodada & D. Selnekovič lgt.”; 3 ♂♂, 4 ♀♀: “Malaysia, Sarawak, Miri distr., Ramudu env., 26.06.2018, (14) 03.54745N, 115.49052E 921 m a.s.l., Pa’Kasi riv., J. Kodada & D. Selnekovič lgt.”; 1 ♂, 4 ♀♀: “Malaysia, Sarawak, Miri distr., Ramudu env., 28.06.2018, (18) 03.54745N, 115.49052E 921 m a.s.l., Pa’Kasi riv., J. Kodada & D. Selnekovič lgt.”; 1 ♀ [FZ1642, MK505418], 1 ♂ [FZ1643, MK505415], 1 ♀: “Malaysia, Sarawak, Miri distr., Pa’Lungan, 30.06.2018, (20) 03.81132N, 115.50737E 1103 m a.s.l., Petarutun riv., J. Kodada & D. Selnekovič lgt.”; 1 ♂ [FZ1635, MK505422]: “Malaysia, Sarawak, Miri distr., Pa’Ukat, 25.06.2018, (13) 03.77346N, 115.47572E 1118 m a.s.l., J. Kodada & D. Selnekovič lgt.”; 1 ♂ [FZ1628, MK505420], 1 ♀ [9, MK505396], 1 ♂ [10, MK505409], 15 ♂♂, 14 ♀♀: “Malaysia, Sarawak, Kuching distr., Bayur riv. near Kampung Bayur, 20.10.2018, 1°14'42.33"N, 110°17'35.26"E 40 m a.s.l., J. Kodada & D. Selnekovič lgt.”; 1 ♀ [FZ1645, MK505404], 1 ♂ [FZ1646, MK505397], 1 ♂ [35, MK505399], 1 ♀ [36, MK566772]: “Malaysia, Sarawak, Kuching distr., Kampong Jangkar env., 10.7.2018, (29) 01.65911N, 109.70829E 67 m a.s.l., J. Kodada & D. Selnekovič lgt.”; 1 ♂ [FZ1636, MK505394]: “Malaysia, Sarawak, Miri distr., Ramudu env., 26.06.2018, (14) 03.54745N, 115.49052E 921 m a.s.l., Pa’Kasi riv., J. Kodada & D. Selnekovič lgt.”. **SABAH**: 50 ♂♂, 46 ♀♀: “Malaysia, Sabah, (Borneo), Kuamut river env. near Kampung Pisang Pisang, 3.-4. VI. 1996, 14a: shaded stream in primary forest with submerged wood”; 12 ♂♂, 4 ♀♀: “Malaysia, Sabah, Kuamut river env. near Kampung Pisang Pisang, 3.-4. VII. 1996, 14b: ca 10 m wide tributary of Kuamut River in primary forest.”; 2 ♂♂, 1 ♀: “Malaysia, Sabah, ca 7 km S Sapulut, Saupi riv. in primary forest, 15.5.2001, J.F. Kočiam leg.”; 1 ex. (sex not examined) [FZ1651, MK505400]: “Malaysia, Sabah, Tawau Division (Kalabakan), 10.7.2018, (MY16-MAL40) 04.560750N, 117.158367E 210 m a.s.l., Čiampor & Čiamporová-Zaťovičová lgt.”; 1 ♂, 2 ♀♀: “Malaysia: Sabah: Maliau Res. Center.: Belian trail; small Maliau R. tributary; bottom gravel, riffle; ca 4°44'15"N, 116°58'15"E, 220 m a.s.l., 27.Sep2017, leg. H. Freitag, C.V. Pangantihon, I. Njunjić et al. / Taxon Expeditions (MRC2c)M”; 2 ♀♀: “Malaysia: Sabah: Maliau Basin: Agatis River; subm. wood, run; ca 4°41'51"N, 116°54'30"E, 520 m a.s.l., 02.Oct2017, leg. C.V. Pangantihon & I. Njunjić / Taxon Expeditions (AgtR2f)M.”. **Brunei**: 1 ♂, 1 ♀: “Brunei: Temburong, Belalong River tributary Sungai Sibut; W of Ashton Trail, root packs, run; 4°32'38"N, 115°08'51"E, 170 m a.s.l.; leg. H. Freitag & W.C. Hayden / Taxon Expeditions 29.Sep2018 (SiCg)M”; 2 ♂♂, 2 ♀♀ [H39, LR735553]: “Brunei: Temburong; Belalong River near UBD field station 4°32'49"N, 115°09'30"E, ca 100 m a.s.l.; primary forest; submerged wood in run; leg. Pangantihon / Taxon Expeditions 28.Sep2018 (BeR1f)M”.

##### Diagnosis.

*Ancyronyx sarawacensis* is a moderately large, usually yellowish species with a pronounced variability of coloration and body size (Figs 3C, D, 4A–D; Tabs 4, 5). From the most similar *A. clisteri* sp. nov., *A. sarawacensis* differs mainly in: 1) anterior elytral portion with expanded yellowish area, much larger than in *A. clisteri* sp. nov.; 2) ovipositor with shorter and wider distal portion of coxite which is ca 1.5–1.6 × as long as wide near middle; 3) proximal portion of coxite subequal in length to distal portion; 4) longitudinal baculum of paraprocts ca 1.3–1.4 × as long as entire coxite length; 5) about 9% divergence of the partial mtDNA for cytochrome c oxidase subunit COI. In the direct comparison the apex of the penis appears to be wider and less pointed in *A. sarawacensis*.

##### Redescription of the holotype.

Body form moderately elongate; elytra moderately convex dorsally, shiny, with highest point in 0.45 of elytral length; BL: 1.45 mm, EW: 0.71 mm, BL/EW: 2.04.

Coloration (Fig. 3C). Labrum, mouth parts, antennae, clypeus and anterior half of frons yellowish; remaining portions of cranium black; pronotum yellowish with large mesal black spot, spot narrowed near middle; scutellum brownish. Elytra with lateral margin and epipleura black along anterior half; dorsum extensively yellowish with six black spots: a small elongate sutural spot behind scutellum; a second, elongate narrow sutural spot on elytral declivity; two lateral, suboval spots extending within elytral striae 3–9 from anterior third slightly behind middle length of elytra; two subapical small spots within elytral striae 4–5. Mesanepisterna, metanepisterna, lateral portion of metaventrite and lateral portions of ventrite 1 black; coxae yellow; femora black on distal sixth, yellowish on remaining surface; tibiae dark in proximal half and near articulation with tarsi, yellowish in distal portion; tarsomeres 1–2 darker; tarsomeres 3–5 and claws yellowish.

Head. Labrum shorter than clypeus; anterior margin almost straight; surface with fine irregular punctation, finely setose. Clypeus wide, densely punctate; sides rounded. Frons and vertex densely, finely punctate, reticulate; reticulation more distinct on black portion of vertex; surface with narrow, elongate distinct granules; frontoclypeal suture almost straight, finely impressed. Eyes well protruding, with large facets, semicircular in outline; HW: 0.38 mm, ID: 0.20 mm. Antennae 11-segmented, 0.46 mm long, moderately longer than pronotum; ratio of length of antennomeres 1–11: 0.055 : 0.061 : 0.042 : 0.030 : 0.031 : 0.030 : 0.029 : 0.029 : 0.032 : 0.037 : 0.089 mm. Gena microsculptured; gula narrow, smooth; gular sutures indiscernible; posterior tentorial pits deep and large.

Thorax. Pronotum distinctly wider than long (PL/MW: 0.81), widest near middle; anteriorly attenuate; anterior margin strongly arcuate; most of hypomeral portion visible in dorsal view; anterior transverse groove deep, oblique, dividing pronotum; area anterior and posterior of transverse groove strongly gibbous; anterior mesal longitudinal carina absent; posterolateral oblique grooves strongly impressed (Fig. 6E, F). Pronotal surface densely punctate and irregularly reticulate on disc, with flat cordiform granules mainly in lateral and anterior portion; granules rather irregularly spaced; anterior and posterior portion smooth; prescutellar pits round and deep; PL: 0.42 mm, MW: 0.52 mm. Prosternum densely and roughly punctate; anterior portion very short; prosternal process distinctly transverse, almost flat, slightly protruding posteriad; posterior margin widely rounded. Scutellum subpentagonal; surface smooth and moderately elevated. Elytra elongate; EL: 1.06 mm; sides slightly narrowed at ca anterior 0.4, then gradually convergent to rounded apices; each elytron with ten more or less regular rows of punctures; six rows between suture and shoulder; accessory scutellary rows absent (Fig. 6C); punctures large, round and deeply impressed on disc and laterally, smaller and less distinct anteriorly and posteriorly; interstices and intervals wider and flat on disc, narrower and slightly convex laterally and posteriorly; surface shiny; humeri prominent. Mesoventrite almost flat, less than half as long as prosternum along midline; mesoventral cavity shallow and narrow; surface strongly and irregularly punctate; mesoventral discrimen invisible. Metaventrite along midline distinctly longer than combined length of prosternum and mesoventrite; anterior margin arcuate; disc with large mesal longitudinal depression; discrimen depressed; surface of disc glabrous, with fine, scarce punctures; large, deep irregular punctures along anterior margin and on lateral portions of metaventrite; punctures coarser and denser laterally (Fig. 6D). Hind wings fully developed. Forelegs about 1.54 × as long as body length; claws large and robust, with one small subbasal and one hardly discernible, basal tooth.

Abdomen. Intercoxal process longer than length of ventrite 2, very wide, anteriorly widely arcuate, with rows of deep large punctures along anterior margin; ventrite 1 longest; ventrite 2 ca 0.75 × as long as ventrite 1; ventrite 3 ca 0.88 × as long as ventrite 2; ventrite 4 ca 0.75 × as long as ventrite 3; ventrite 5 ca as long as combined lengths of ventrites 3 and 4. Surface of ventrites 2–5 with sparse punctures and setigerous, flat, more or less cordiform granules; punctures more distinct on mesal portion, granules more prominent and more conspicuous laterally. Male sternite IX ca 340 μm long, asymmetrical; apical margin distinctly arcuately emarginated; lateroapical portion with short setae; paraprocts not reaching beyond apical margin. Tergite VIII finely reticulate, with conspicuous median transverse ridge separating posterior and anterior portion; basal half with microtrichial pattern; apical margin hyaline, with subapical fringe of hair-like setae; setae on sublateral portions stronger and longer than those along margin.

Aedeagus (Figs 15A, B, 16A, B) ca 358 μm long; penis about 2.63 × as long as phallobase; sides subparallel from base to apical fourth then gradually tapering apicad; apical half moderately curved ventrad; dorsolateral portion only with few very short setae; apex narrowly rounded; lateral basal apophyses long; ventral sac large; fibula long and narrow; surface of endophallus with numerous almost regularly arranged, small spines; corona not discernible in endophallus repose. Entirely extruded endophallus more than twice as long as aedeagus, asymmetrical (description based on one specimen (CKB) from the type locality); most of the following characters of the endophallus, even if it is extruded, are difficult to observe and clearly visible in Fig. 10G: supporting sclerites of endophallus thin; ventral and dorsal bladders bulbous, extending less than half of penis length, with few very fine short thin setae; distal portion of endophallus tubular, very long; apex narrowed and supported by a fine elongate sclerite near gonopore (Fig. 10G). Phallobase asymmetrical; parameres moderately exceeding midlength of penis, widest near base, narrowed to apex; dorsal and ventral margin sinuate; apex narrowed and rounded; outer surface of parameres with short setae.

##### Description of ovipositor

(Fig. 17A–C). Ovipositor in females from the type locality ca 435 μm long; stylus (gonostylus) narrow, straight, about 0.7 × as long as distal portion of coxite. Coxite short and stout; posterolateral angle rounded, not protruding. Distal portion of coxite ca 1.5–1.6 × as long as wide, moderately bent; surface with numerous conspicuous peg-like/spine-like setae; apical area with few moderately long trichoid and few thinner peg-like setae; inner margin moderately pubescent. Proximal portion of coxite about as long as distal portion, with numerous stout peg-like and trichoid setae; transverse baculum well sclerotised; longitudinal baculum of paraprocts ca 1.3–1.4 × as long as coxite length.

##### Secondary sexual dimorphism.

Not strongly pronounced. On average, females are longer and wider than the males (Tabs 4, 5). Females possess narrower and shallower longitudinal depression of the metaventrite, and their terminal ventrite is longer and narrower than in males. All these differences named here refer to syntopic specimens.

##### Variability.

In addition to the metric characters (Tabs 4, 5), adults of *Ancyronyx sarawacensis* vary in color pattern, body form and surface structures. This is obviously dependent on altitude and water temperature. Specimens from the Kelabit Highlands usually exhibit a more extensively black cranium, their pronotal spot is usually not or less distinctly divided, the elytral spots are wide (Fig. 3D). Generally, any fusion of elytral spots is rather rarely seen and if, then only the three posterior spots fuse. The elytral surface appears shinier and smoother; the anterior transverse pronotal groove is deep, and areas anterior and posterior of these grooves are strongly gibbous, and their body form appears generally shorter and more convex. In comparison, the specimens from Sarawak and Brunei lowlands (Gunung Mulu NP, Kapit area, Kampong Bayur, Kampong Jangkar, Temburong) usually have a paler cranium, and their pronotal spots are more distinct, smaller and isolated (Fig. 4B–D). Furthermore, their elytral surface is less shiny, and their interstices are narrower, the pronotum is almost flat and the body appears overall slenderer. Specimens from Sabah uplands (Figs 3D, 4A) tend to have darker heads and pronota and separately fused large anterior and posterior elytral spots, their color pattern is very similar to those of *A. clisteri* sp. nov. Pronota of these specimens are usually more convex, similar to those of the specimens from the Kelabit Highlands.

##### Material examined. Larva.

Nine larvae of three different sizes/instars including the larva used in the DNA analysis (CKB): “Malaysia, Sarawak, Miri distr., Bario env., 5.10.2018, (33) 3.759617N, 115.440233E 1143 m a.s.l., Arur Dalan, J. Kodada & D. Selnekovič lgt”. For the description, the two largest larvae, probably representing last instars, were selected.

##### Matching of developmental stages.

All larvae were collected together with adults at the type locality. One larva [FZ1631, MK505395] was compared with adults of *A. sarawacensis* based on partial COI mtDNA sequences.

##### Diagnosis of mature larva

**Figure 7.**
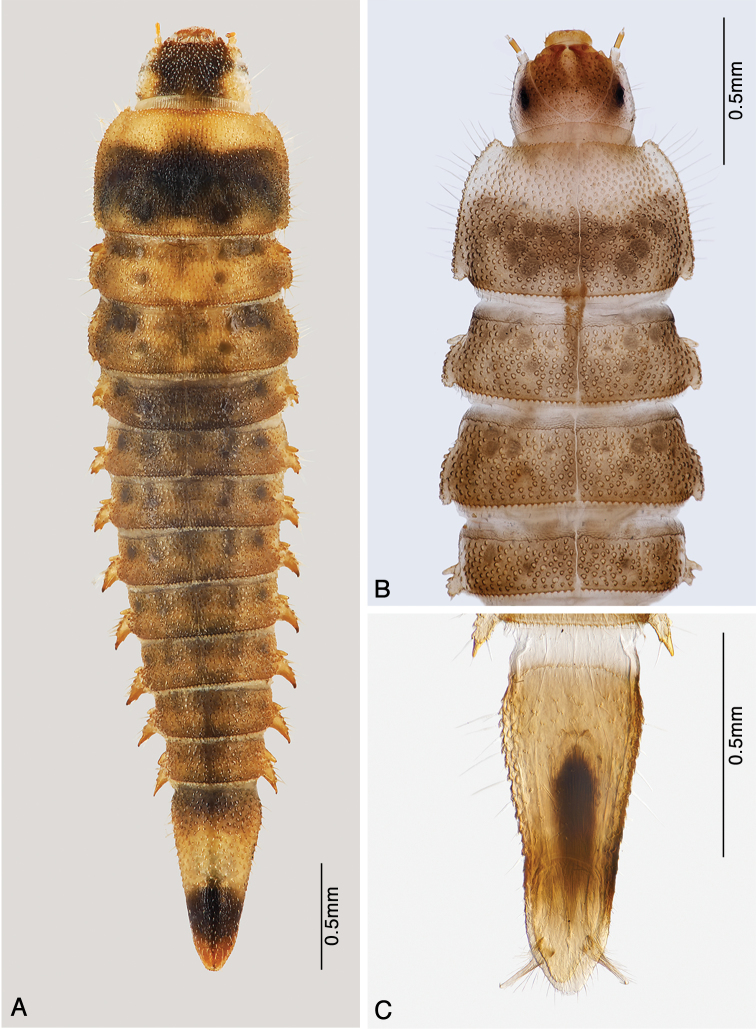
*Ancyronyx sarawacensis*, larvae from the type locality: **A** final instar, dorsal view **B** pre-final instar, cleared specimen mounted on microscopic slide, detail of anterior portion, dorsal view **C** same specimen, terminal abdominal segment showing hooks of operculum, ventral view.

**Figure 8.**
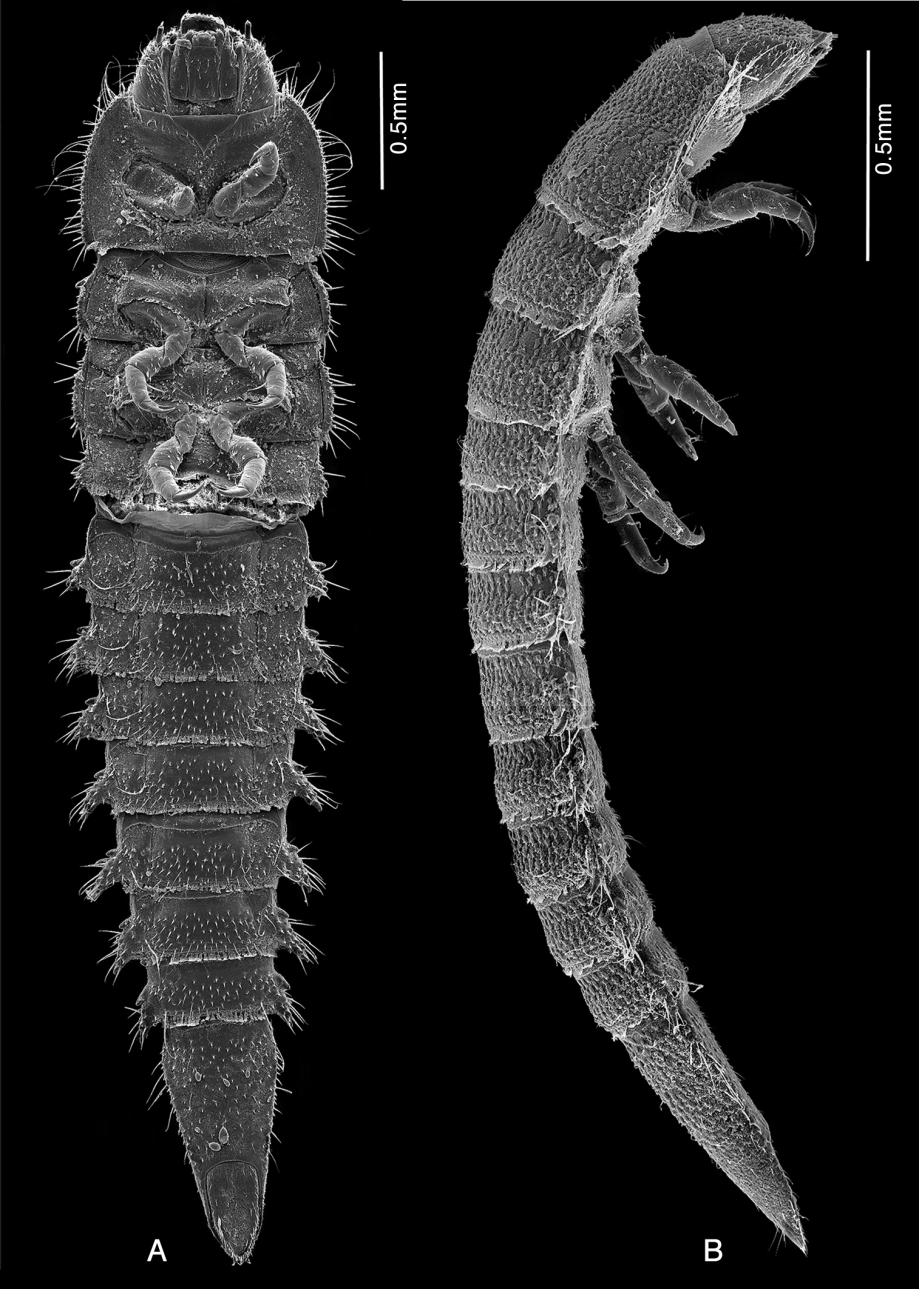
*Ancyronyx sarawacensis*, larvae from the type locality, SEM micrographs: **A** final instar, ventral view **B** pre-final instar, lateral view.

**Figure 9.**
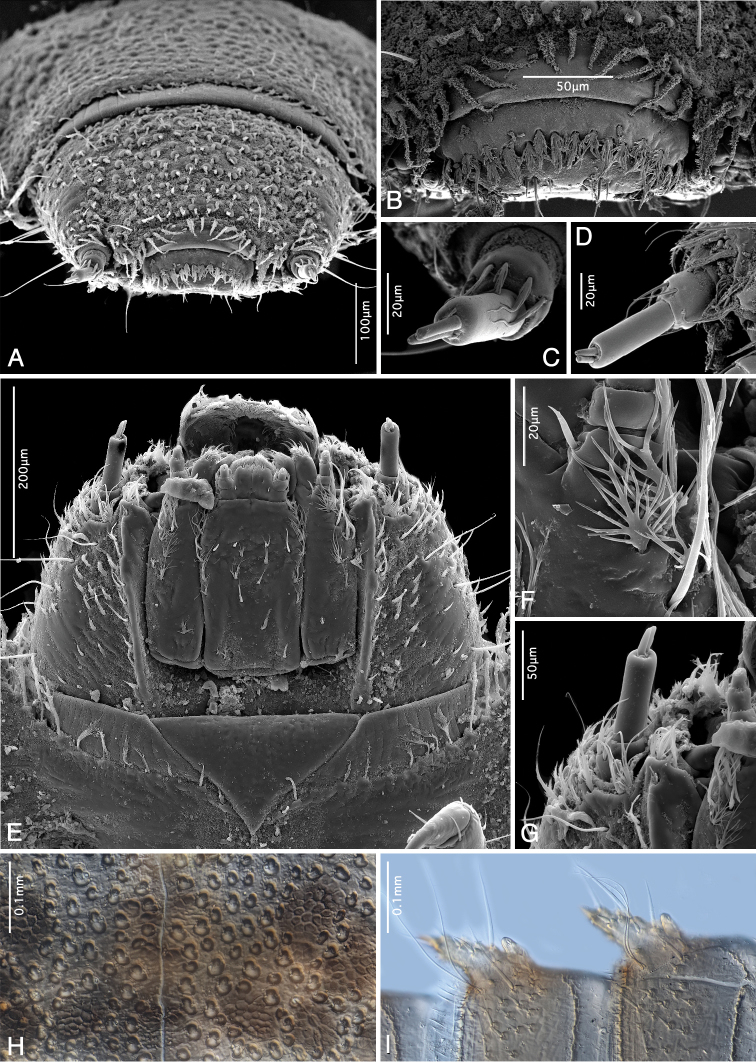
*Ancyronyx sarawacensis*, larvae (pre-final instar) from the type locality, SEM micrographs and cleared microscopic slide mounts: **A** head, frontal view **B** labrum, frontal view **C** left antenna, frontal view **D** right antenna, in rotaded view **E** final instar, head, ventral view **F** detail of ramified setae on anterior portion of stipes, ventral view **G** antenna, ventral view **H** detail of surface structure of prothorax, dorsal view **I** lateral portion of abdominal segments VI and VII, ventral view.

**Figure 10.**
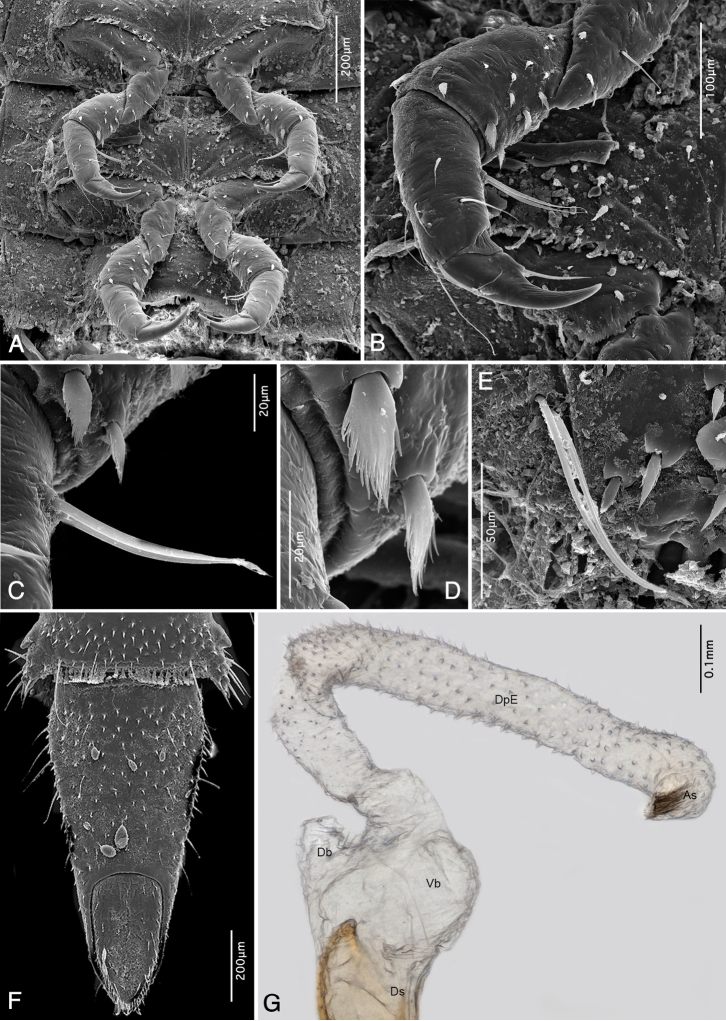
*Ancyronyx sarawacensis***A–F, H** final instar larvae from the type locality, SEM micrographs and cleared microscopic slide mounts, **G** everted endophallus: **A** meso- and metathorax with legs, ventral view **B** right middle leg, detail, ventral view **C** detail of twin setae on tibiotarsus, ventral view **D** detail of pilose setae on femur, ventral view **E** detail of twin setae on pleurite VI, ventral view **F** terminal abdominal segment, ventral view **G** extruded endophallus, dorsolateral view. Abbreviations: Ds: dorsal sac-supporting sclerite, Vb: ventral bladder, Db: dorsal bladder, DpE: distal portion of endophallus, As: apical sclerite (according to Hayashi and Yoshitomi 2015).

(Figs 7A, 8A, 9E–G, 10A–F, H). Body elongate, tapered dorsad; length from anterior margin of head to apex of abdomen: 4.43 mm; largest width across metanotum: 1.01 mm; body ventrally almost flat, dorsally convex; dorsal sagittal line present from prothorax up to sixth abdominal segment (Fig. 7A). Ratio of length of thoracic segments and abdominal segment IX: 0.60 : 0.35 : 0.32 : 0.84 mm; lengths of all remaining abdominal segments between 0.21 and 0.26 mm. Thoracic segments II and III with flattened lateral tergal processes (Fig. 7B); abdominal segments I–VIII with conical prominent bent lateral tergal processes (Figs 7A, 9I). All spiracles small and subequal in form and size, biforous, situated at ends of prominent spiracular tubes on mesothorax and abdominal segments I–VIII (Figs 7A, B, 9I). Dorsum densely covered with flat setiferous tubercles except for rugose distal portion of terminal segment (Fig. 7B). Antennae and legs very short (Fig. 8A, B). Prevailing ground color (Fig. 7A) yellowish-brown combined with dark brown patterns on frons, adjacent portion of epicranium, posterior two thirds of pronotum; meso- and metanotum each with four pairs of dark brown signae (Fig. 7B); genae very pale, almost white; abdominal segments I–VIII with two dorsolateral pairs of dark brown signae and with brownish spot near middle, dark coloration increasingly inconspicuous posteriorly (Fig. 7A); segment IX pale brown on anterior portion, dark brown apically (Fig. 7A). In contrast to previous darker patterns, anterior third of pronotum, dorsolateral portions of cranium, middle portion of abdominal segment IX as well as appendages and entire venter paler yellowish.

Head prognathous, partly retractable, distinctly narrower than pronotum (Fig. 9A); maximum head width 0.55 mm. Labrum very short, ca 2.5 × as wide as long, separated from head capsule by complete suture, anterior margin arcuate, middle with transverse row of moderately long, ramified setae (Fig. 9B); clypeus subequal in length with labrum, with a row of pilose setae along posterior margin; frontoclypeal suture complete. Anterior margin of frons arcuate, with a row of moderately long, pilose setae; anterolateral angles form short conical projections, each projection with one distinct peg-like pilose seta. Frontal arms broadly V-shaped, well impressed, epicranial stem absent (Fig. 7B); surface of frons and adjacent area of epicranium with cordiform tubercles, which are distinct, flattened and provided with short scale-like setae (Fig. 9A); anterolateral portion with one moderately long trichoid seta. Setal pattern on epicranial plates: one moderately long trichoid seta near dorsal margin of stemmata; one at 0.6 of cranial length near frontal arm; one ventral near antennal insertion; four long and very conspicuous trichoid setae on lateroventral portion; several simple spine-like setae on lateral/laterodorsal portion, mostly widely spaced, four of them arranged in an oblique laterodorsal row; genae with several spine-like slightly serrate/pilose setae on ventral portion. Antennae (Fig. 9C, D, G) three-segmented, short, approximately 0.25 × as long as maximal head width. Scape (Fig. 9C, D) elongate, moderately longer than wide, with three moderately ramified setae around distal margin (two trichoid setae of varying length and a single, moderately long, pointed peg-like seta); pedicel narrow, cylindrical and about 1.5 × as long as scape, distally with two minute peg-like setae (Fig. 9G); flagellum and sensorium subequal in length, both elongate and about 4 × as long as wide; terminal segment with minute setae. Stemmata arranged in a single unified lateral spot, not exposed (Fig. 9A). Mandibles (Fig. 13C) short, distinctly longer than wide, left and right mandible almost symmetrical; apices wide, tridentate, ventral and dorsal teeth continuing in ventral and dorsal carina; right mola convex and prominent, left mola flat, asperities absent; articulated process setose, robust, half as long as mandible. Penicillus with moderately long, thin, setae, densely arranged in a row along mesal portion; outer mandibular edges arcuate, each bearing four conspicuous ramified setae; ventral condyles robust and prominent.

Ventral side of cranium (Fig. 9E) with strong hypostomal ridge extending posteriad into robust hypostomal rods; the latter exceeding middle of cranium; oblique row of 3–5 short stout peg-like setae situated posteriad of hypostomal rods. Posterior tentorial pits small, gular sutures absent. Maxilla elongate, large, almost half as wide as mentum (across stipes); cardo very short, transverse, almost perpendicular to stipes. Stipes elongate, with several different setae (Fig. 9F): one short ramified seta near base; four moderately long, extensively ramified setae in a submarginal row along anterior half; few conspicuous long trichoid setae on anterior fourth; palpifer well sclerotised, short and wider than palpomeres, apical portion with two spine-like setae. Maxillary palp three-segmented (Fig. 9E), distinctly shorter than stipes width; palpomeres with a few short trichoid setae, terminal palpomere with several small peg-like sensilla. Galea undivided, elongate, about 2 × as long as wide; apex narrowed and rounded, with row of several moderately bent spines and numerous short, mesally directed setae; dorsal surface with cluster of dense thin setae. Lacinia subequal to galea in form and length; apex with dense, strong spines. Labium with very short and transverse submentum oriented vertically to mentum. Mentum (Fig. 9E) about 1.6 × as long as wide; widest at anterior third; median portion shallowly impressed, laterally with row of six moderately long, extensively ramified setae in anterior 0.6 and a single moderately long trichoid seta at anterior third; anterolateral portion with one distinct stout peg-like seta on each side; disc of mentum with pair of mesal, longitudinal rows of five trichoid and ramified setae. Ligula with mesal longitudinal line, about as long as lacinia, with four short peg-like setae; anterior margin arcuate and densely setose; labial palp very short; apical segment similar to that of maxillary palp; palpiger undifferentiated.

Pronotum (Fig. 7A, B) 1.5 × as wide as long; posterior half with seven pairs of partly fused small and rather inconspicuous areas (signa) with irregular polygonal reticulation (Fig. 9H); three pairs of erected trichoid setae present on disc and one pair on posterolateral position; lateral margins fringed with long trichoid setae. Meso- and metanotum more than 3 × as wide as long, distinctly shorter than pronotum, each with five pairs of partly fused signa and one posterolateral and two admedian pairs of long erected trichoid setae; sides projected laterally, fringed with long setae; surface with tubercles. Posterior margins of all nota dentate, fringed with row of scale-like setae. Ventral side of prothorax with small triangular presternum (Fig. 8A) and three hardly identifiable sclerites: one large anterolateral sclerite on each side and one small transverse sclerite on posteromesal portion; sclerites completely surrounding coxae, connate, more or less discernible in cleared specimen only. Ventral portions of both, meso- and metathorax, with six sclerites (Figs 8A, 10A): two large anterior sclerites divided by fine line mesally; each side with one small anterolateral sclerite and one large lateral sclerite; the latter projecting moderately at posteromesal portion; anteromesal sclerites with rows of short spine-like setae along posterior margin. Legs (Figs 8A, B, 10A) stout, five-segmented, about 0.5 × as long as thorax width, all similar in shape and length. Coxae large, distinctly wider than long, subellipsoidal; mesal portion moderately projecting ventrad; surface with a few short setae. Trochanters subconical, each with a single long trichoid seta on distal third. Femora subcylindrical with scattered short peg-like setae, most of them with serrate/setose margins (Fig. 10D, E); largest setae concentrated on inner portion and around distal margins. Tibiotarsi subcylindrical, narrower than femora, subequal in length with trochanters and femora; surface with several different trichoid setae in distal portion and a pair of distinct, very closely set trichoid seta (Fig. 10B, C). Pretarsi elongate, moderately bent, each with a single long trichoid seta.

Abdominal segments I–VIII similar in shape, each distinctly wider than long, sub-rectangular; sagittal line visible in segments I–VI; lateral tergal processes of segments I–VIII conical, prominent and bent with dorsally directed pointed tip (Figs 7A, 8A); surface with asperities, stout setae and short spines; processes of subsequent segments moderately increase in size. Posterior margins of terga dentate and each fringed with scale-like setae. Ventral portion of segment I–VII with three well differentiated sclerites; median sclerite (sternite) of segment I widest, about 2.8 × as wide as a lateral sclerite (pleurite); sternites moderately narrowed posteriad, pleurites distinctly narrowed posteriad; pleurites of segments VIII–IX completely fused with tergites and sternites. Ventral surface with numerous stout short spines and several longer thinner trichoid setae; the latter concentrated on posterolateral portion; segments I–VIII with a pair of long, very closely set trichoid setae near basis of lateral process (Figs 9I, 10F); posterior margins of segments dentate and fringed with scale-like setae. Terminal segment (Figs 7A, C, 10F) 2.1 × as long as wide, widest at anterior 0.15, subconical; subtriangular in cross-section; apex narrowed. Dorsal surface with flat setiferous tubercles; the latter large and distinct on anterior two thirds, absent or inconspicuous on posterior third; setae on tubercles short; setae along lateral margins long and very distinct; several moderately long erect-trichoid setae intermixed within granules on admedian portion. Ventral side with scattered, short or moderately long trichoid setae. Operculum (Fig. 10F) elongate, subtriangular, almost 2 × as long as wide, slightly impressed medially, rugose; lateral and apical margins with trichoid setae; internally inserted pair of hooks half as long as operculum (Fig. 7C); each hook with two clusters of longitudinally arranged trichoid setae near base.

##### Variation between larval instars.

The pre-final instars are shorter (3.85 mm) with narrower head (HW: 0.43 mm) and exhibit more evenly brown color from the posterior pronotal portion up to the anterior portion of segment IX. The pale anterior portion on the pronotum extends up to half of the pronotal length. Segment IX is relatively slender and 2.3 × as long as wide vs. ratio 2.1 in the final instar. The triangular presternum is not yet clearly delimited.

##### Comparative remarks.

The body shape of the larva is typical for the *Ancyronyx variegatus* (Germar) species group. In this group, larvae were described for *A. helgeschneideri* Freitag & Jäch, *A. procerus*, *A. schillhammeri* Jäch and *A. variegatus* (Brown 1972; Freitag and Balke 2011; Freitag 2013). In contrast to those of the *A. patrolus* species group, these larvae share the comparably large size, dorsoventrally depressed body, stout legs and prominent conical lateral tergal processes (posterolateral appendages). *Ancyronyx sarawacensis* closely resembles *A. helgeschneideri* (comp. Freitag and Balke 2011) in the yellowish-brown color, similar size (final instar HW 0.55 mm vs. 0.50 mm; body length 4.4 mm vs. 4.5 mm), relatively large spiracles, similar shape and distribution of setae, tubercles and asperities, similar proportions and color pattern of abdominal segment IX, arcuate sides of head, and presence of short anterolateral frontal projections and lack of a median frontal projection. *Ancyronyx sarawacensis* differs from *A. helgeschneideri* in the pale anterior third of pronotum (vs. pale anterior and lateral pronotal margins), labrum with transverse row of ramified setae (vs. tuberculate with rather inconspicuous ramified and trichoid setae), submentum relatively longer and slenderer (1.6 × as long as wide vs. 1.4 ×) and clearly delimited triangular presternum. Larvae of *Ancyronyx procerus* (comp. Freitag and Balke 2011) can be distinguished by head being almost as wide as pronotum with sides almost straight (vs. head distinctly narrower than pronotum with sides arcuate), presence of a median frontal projection and three prominent frontal projections (vs. short anterolateral frontal projections). Furthermore, *A. sarawacensis* larvae are relatively slenderer than those of *A. procerus* (final instar HW 0.55 mm vs. 0.62 mm, body length 4.4 mm vs. 3.7 mm) and dorsally more densely covered with larger tubercles.

##### Habitat.

**Figure 11.**
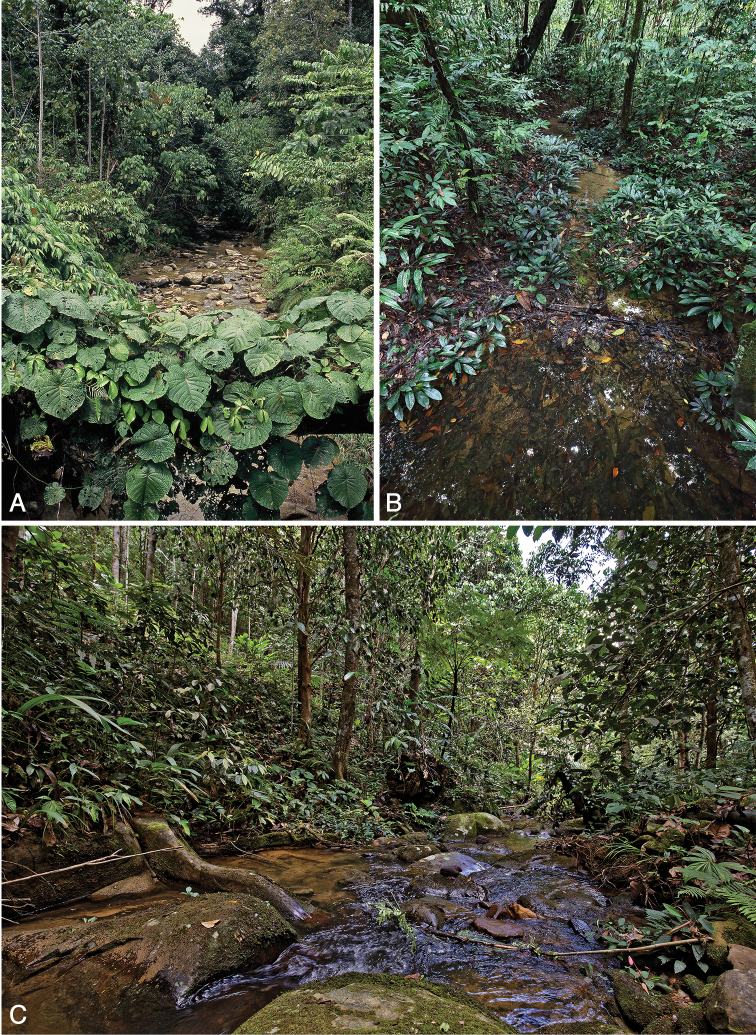
Habitats of *Ancyronyx clisteri* sp. nov. and *A. sarawacensis*: **A** type locality of *A. clisteri* sp. nov., tributary of the Kuamut River near Kampung Pisang Pisang, Sabah **B** atypical locality of *A. sarawacensis*, shaded shallow stream in primary forest, Gunung Mulu NP, Sarawak **C** type locality of *A. sarawacensis*, small stream above Arur Dalan near Bario, Kelabit Highlands, Sarawak.

The altitude of all collection sites ranges from 40–1200 m a.s.l; the species is described from a small stream near Arur Dalan in the Kelabit Highlands (1000–1200 m a.s.l.). It is an upper reach (epirhithron), ca 3 m wide, with boulders and cascades, flowing through degraded primary forest. Water quality is presumably very good since the stream serves as drinking water for the settlements. Current and bottom substrates are heterogeneous; the latter includes mineral and organic deposits. All *Ancyronyx* specimens were collected from submerged wood. The larvae were found together with adults hidden in fissures of the bark of a large, relatively fresh submerged tree branch fully covered with bark. Mountain streams in the Bario area (Pa’Ramapoh, Arur Takang and Pa’Marario) have fewer boulders, fewer cascades and more submerged wood. The river at Pa’Ukat is ca 10 m wide, shallow, slowly flowing, entirely shaded, with stony and sandy substrates and numerous fallen trees. Forest streams near Ramudu, Pa’Ngaruren and Pa’Kasi are ca 4–7 m wide, slowly flowing and meandering, with stones, some cobbles and sand substrates, submerged woods, leaf packs and exposed roots. Specimens were collected mostly from submerged dead wood, however, a few specimens come also from submerged roots of tree or bamboo. The Petarutung River in Pa’Lungan is 7–10 m wide, shallow, meandering, with sandy bottom and represents the only reddish colored river where some specimens were found (water color is probably due to humic substances from a nearby peat swamp forest). Outside the Kelabit Highlands *A. sarawacensis* was collected in small lowland and upland forest streams, which are moderately wide, rather shallow, usually meandering and with sandy or stony substrates, always containing submerged logs, woody debris and leaf packs. The specimens were collected mainly, but not exclusively, in stream reaches with stronger currents. The most atypical stream inhabited by *A. sarawacensis* was a small, slowly flowing, very shallow, meandering creek in Gunung Mulu NP (55 m a.s.l.) with large amounts of accumulated leaves and a sandy bottom, with some gravel and a few submerged branches (Fig. 11B); specimens were collected together with *Ancyronyx pulcherrimus* Kodada, Jäch & Čiampor and a new species of *Okalia* Kodada & Čiampor.

**Figure 12.**
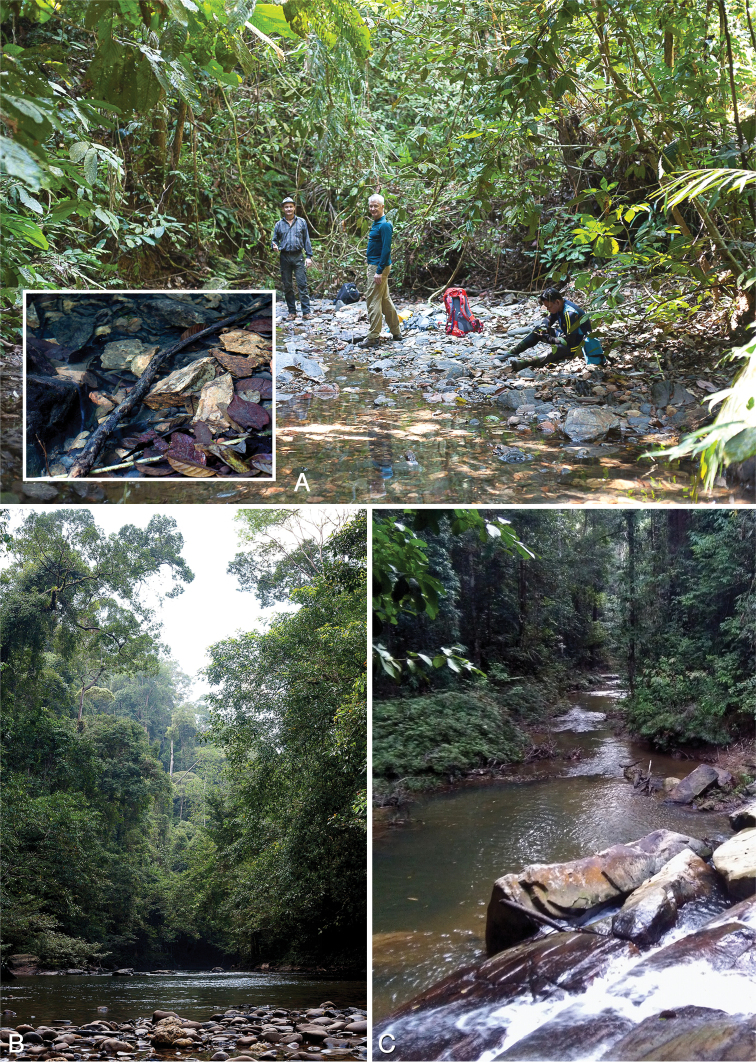
Habitats of *Ancyronyx clisteri* sp. nov. (**A**) and *A. sarawacensis* (**B, C**) sampled during Taxon Expeditions: **A** Sibut Creek (tributary of Belalong River), Temburong, Brunei, microhabitat (piece of submerged wood from which *A. clisteri* sp. nov. was collected) **B** Belalong River near Kuala Belalong Field Studies Centre, Temburong **C** Agatis River in the vicinity of Maliau Basin, Sabah, Malaysia. All photographs by Clister V. Pangantihon.

**Figure 13.**
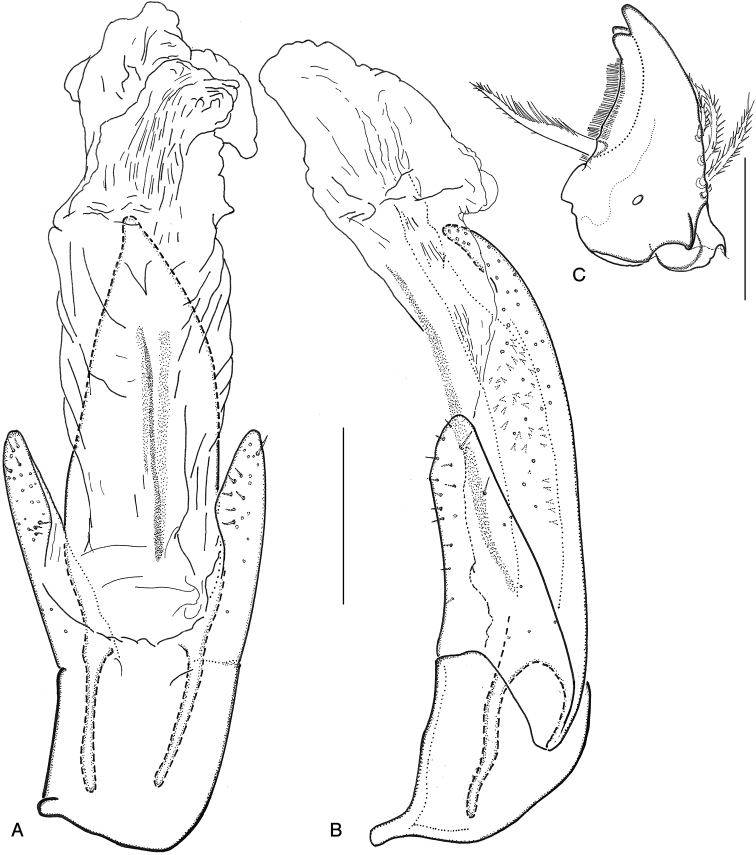
**A***Ancyronyx clisteri* sp. nov., aedeagus of holotype with endophallus partly extruded, ventral view **B** same, lateral view **C***A. sarawacensis*, larval mandible, ventral view.

**Figure 14.**
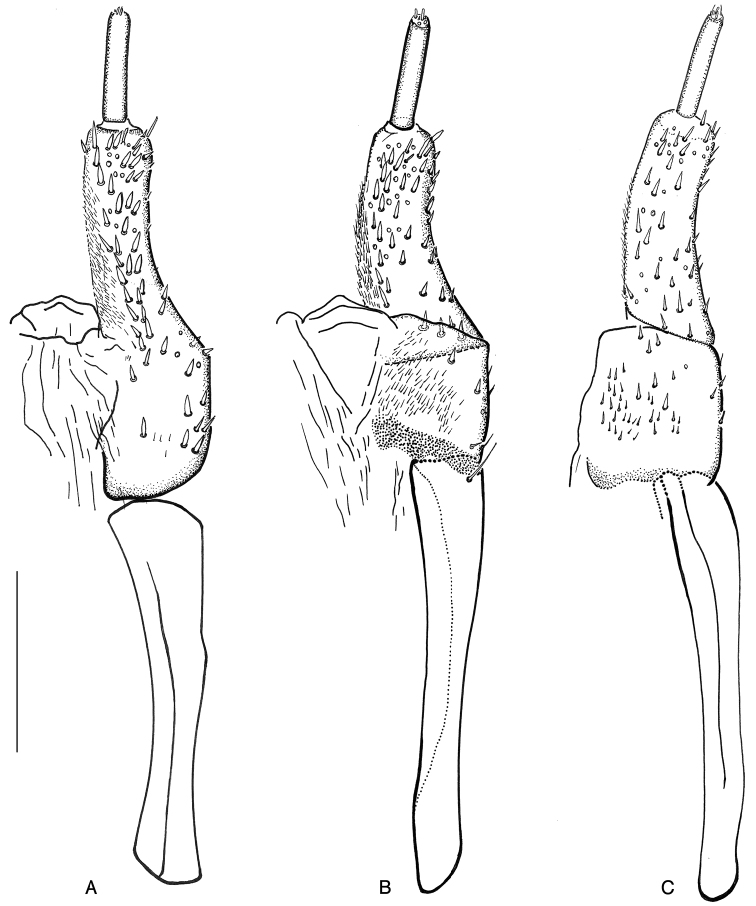
*Ancyronyx clisteri* sp. nov.: **A** ovipositor, paratype from Sarawak, dorsal view **B** same, ventral view **C** ovipositor, paratype from the type locality, ventral view.

**Figure 15.**
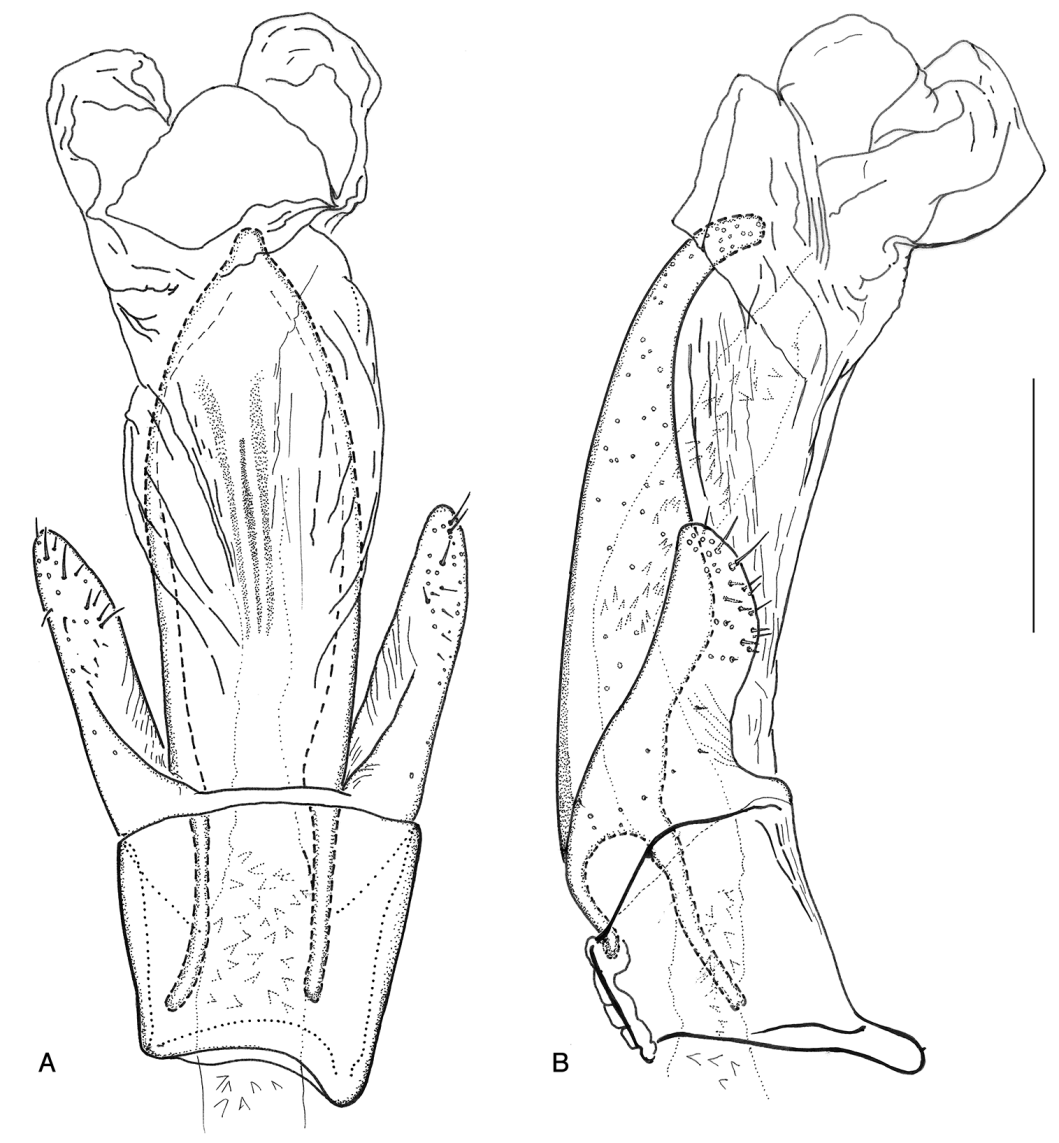
*Ancyronyx sarawacensis*, specimen from the type locality: **A** aedeagus with endophallus partly extruded, ventral view **B** same, lateral view.

**Figure 16.**
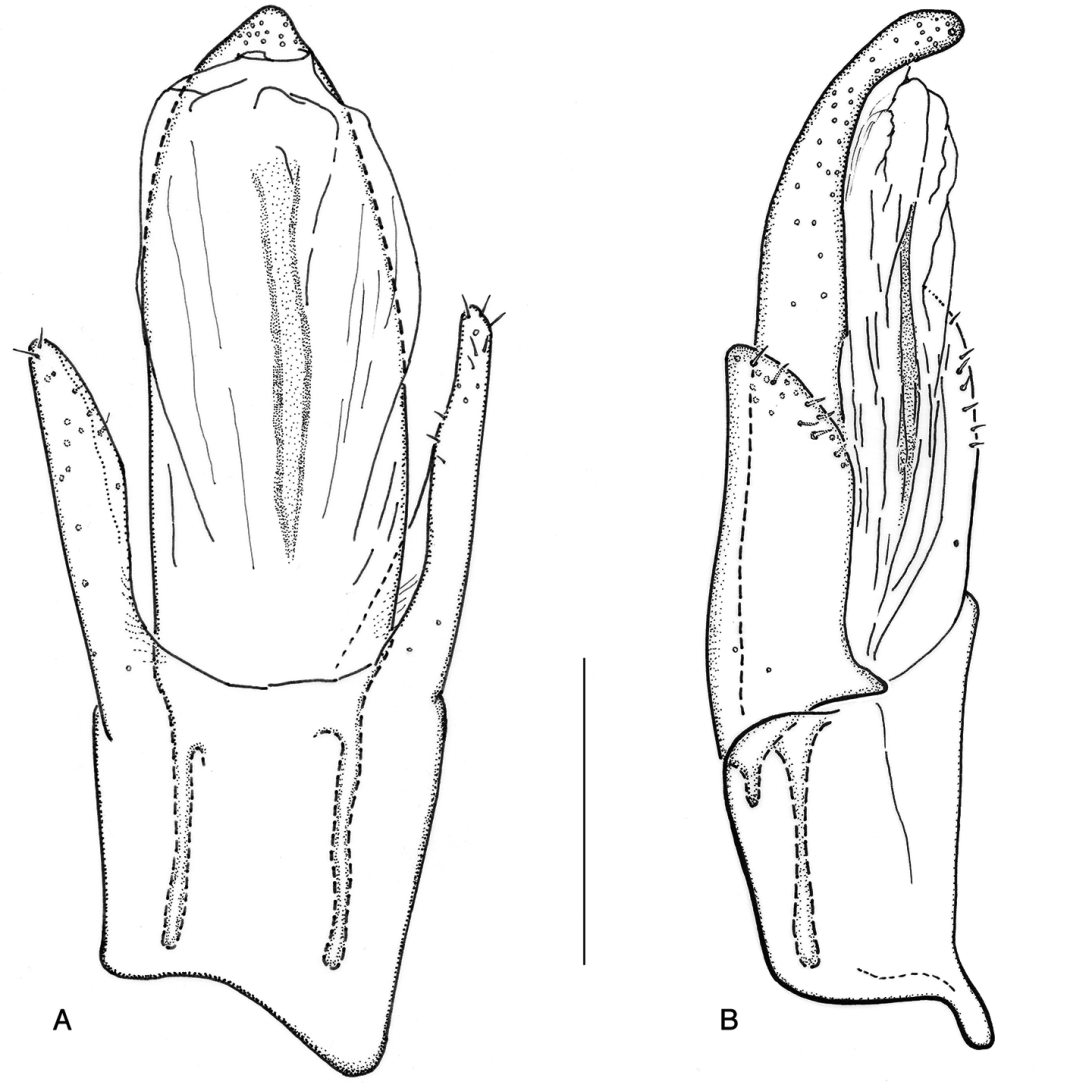
*Ancyronyx sarawacensis*, holotype: **A** aedeagus, ventral view **B** same, lateral view.

**Figure 17.**
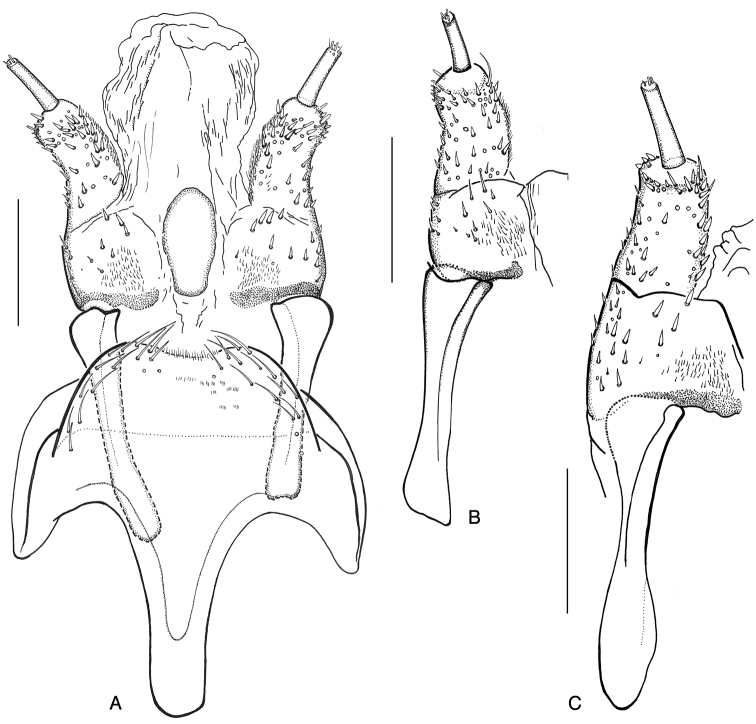
*Ancyronyx sarawacensis*: **A** ovipositor and abdominal segment VIII, specimen from a tributary of Kuamut River, Sabah “(14B)”, ventral view **B** ovipositor, paratype from Kapit area, Sarawak, ventral view **C** ovipositor, paratype from the Kelabit Highlands, Sarawak, ventral view.

##### Syntopic taxa.

Usually, the same piece of submerged wood, especially larger pieces can be inhabited by several genera of Elmidae and Dryopidae. *Ancyronyx sarawacensis* was found with specimens of *Graphelmis berbulu* Čiampor, *G. labralis* Čiampor and *G. mumini* Čiampor at the type locality. From lowland and upland Sarawak and Sabah, the following species were found to be syntopic: *Ancyronyx procerus*, *A. acaroides*, *A. pulcherrimus*, *Graphelmis gemuk* Čiampor and several species belonging to the *G. pict*a and *G. marshalli* groups. Some species of *Leptelmis* Sharp as well as *Stenomystax montanus* Kodada, Jäch & Čiampor, *S. depressus* Kodada, Jäch & Čiampor and *S. minutus* Kodada, Jäch & Čiampor and some species of *Elmomorphus* Sharp were also found syntopic.

##### Distribution.

This species is widely distributed in northern Borneo (Sarawak, Sabah and Brunei). In Sarawak it was collected in several small tributaries of the Dapur and the Kelapang rivers in the Kelabit Highlands; small tributaries of the Tutoh and the Melinau Paku rivers in and near the Gunung Mulu National Park; tributaries of the Sut River near Kapit, the Jangkar River near Lundu and a small stream near Kampung Bayur (Kuching area). In Sabah, it is known from the tributaries of Sapulut River near Batu Punggul; small tributaries of the Kuamut River as well as the Agatis River and a small tributary of the Maliau River. In Brunei, the species was collected from the Belalong River and from one of its small tributaries.

#### Ancyronyx procerus

Taxon classificationAnimaliaColeopteraElmidae

Jäch, 1994

F3330E23-B81D-5558-98DD-02EB1CC2459B

##### Type locality.

Tributary of the Tutoh River, near Long Iman, Mulu National Park, northern Sarawak, Borneo, East Malaysia.

##### Material examined.

***Holotype*** ♂ (NMW): “Malaysia, Sarawak, Mulu NP, Long Iman 4.3.1993 leg. M. Jäch (20)”. ***Paratypes***: 5 exs with same data as holotype (NMW), 2 exs (CKB): “Malaysia - Sarawak ca 40 km E Kapit III. 1994 leg. J. Kodada \ Rumah ugap Ng marating Kapit Sut”.

##### Additional material

(CCB, CFDS, CKB, NMW). **SARAWAK**: 1 ♀ [37, MK505417], 1 ♀ [FZ1639, MK505411], 2 exs: “Malaysia, Sarawak, Kuching distr., Kampong Jantar env., 10.7.2018, (29) 1.65911N, 109.70829E, 67 m a.s.l., J. Kodada & D. Selnekovič lgt.”; 1 ♀ [11, MK505423], 1 ♂ [FZ1644, MK505410], 2 exs: “Malaysia, Sarawak, Kuching distr., Bayur riv. near bridge of jalan Jambur - Bayur, 20.10.2018, 1°14'43.2"N, 110°17'34.8"E, ca 50 m a.s.l., J. Kodada & D. Selnekovič lgt.”; 3 exs: “Malaysia, Sarawak, Miri distr., Ramudu env., 5.03.2019, (No. 51), Ramudu riv., 3°32'14.390"N, 115°30'22.456"E, ca 900 m a.s.l., J. Kodada & D. Selnekovič lgt.”; 4 exs: ”Malaysia, Sarawak, Miri distr., Ramudu env., 6.03.2019, (No. 52), Pa‘Masia riv., ca 3°31'57.736"N, 115°30'39.624"E, ca 950 m a.s.l., J. Kodada & D. Selnekovič lgt.”; 10 exs: “Malaysia, Sarawak, Miri distr., Dapur riv. near Pa’Umor, 8.03.2019, (No. 54), 3°44'3.49"N, 115°30'56.24"E ca 1070 m a.s.l., J. Kodada & D. Selnekovič lgt.”. **SABAH**: 1 ♂: “Malaysia, Sabah, ca 25 km Sapulut, Sabalangang river, 21.V.2001“; 1 ♂, 3 ♀♀, 4 exs: “Malaysia, Sabah, (Borneo), Kuamut river env. near Kampung Pisang Pisang, 3.-4. VI. 1996, 14a: shaded stream in primary forest with submerged wood”; 1 ♂, 1 ex.: “Malaysia, Sabah, Kampung Pisang Pisang env., tributary of Kuamut River, 29.6.1998, J. Kodada & F. Čiampor Lgt.”; 1 ex. [FZ1660, MK505403], 1 ♂: “Malaysia, Sabah, Interior Division (Nabawan), Batu Punggul env., 23.5.2001, Čiampor lgt.”. **West Malaysia: PAHANG**: 1 ex. [FZ1659, MK505405], 8 ♂♂, 3 ♀♀: “Malaysia, Malaysia, Pahang, Kenong Rimba Park, Kesong River, 5.6.2001, J. Kodada & F. Čiampor”. **TERENGGANU**: 4 exs [FZ1623, MK505419; FZ1624, MK505413, FZ1625, MK505402; FZ1626, MK505412]: “Malaysia, Terengganu, Kg. Pancur Merah env., stream ca 7m wide, 5°33'28.86"N, 102°40'47.70"E, 2.8.2016, ca 30 m a.s.l.”.

##### Variability.

*Ancyronyx procerus* represents the largest species of the genus (TL 2.4–2.8 mm) with two, more or less distinct color forms. The form with lighter (less black) elytra and sometimes smaller to obsolete femoral spot correspond in habitus to the type specimens from Long Iman, Sarawak (see Jäch 1994: fig. 28). These lighter specimens were found, e.g., in all Sarawak lowland localities and in Sabah, in the Sabalangang River and in the tributary of the Kuamut River. The darker form with extensive black elytra and darker femoral spot were found in Sarawak in the rivers of the Kelabit Highlands, Sabah and in localities from West Malaysia. These color forms do not represent separate taxa, neither according to their genetic differentiation in COI mtDNA sequences, nor according to the fine differences in their genital morphology.

##### Habitat.

All *Ancyronyx procerus* were sampled from submerged wood and seem to prefer larger pieces of fresh wood with bark; so far none of the specimens were found on submerged exposed roots of living trees or bamboo like adults of *A. sarawacensis* or *A. acaroides* (Kodada and Selnekovič pers. obs.). Adults have also not been collected in light traps yet. The larva was described after two specimens, one from type locality and one from Busuanga Island (Philippines); association with adults was corroborated by COI mtDNA sequences (Freitag and Balke 2011).

Specimens were usually found in smaller shallow meandering rivers with low vertical gradient, or – less frequently – in lowlands streams/creeks with a rather slower current, mostly with gravel or sandy substrate (e.g., Kesong River, rivers near Kampung Jalan and Kampung Bayur, Sabalangan River). In mountainous habitats the species is newly recorded from rivers in the Kelabit Higlands: Dapur, Ramudu and Pa’Masia; all these localities are within altitudes of 900–1070 m a.s.l. The largest of these rivers is the Dapur River; at the collecting place near Pa’Umor it is about 10–15 m wide, deep, reddish colored, meandering, with sandy bottom, and contains lot of submerged logs; the water level is strongly fluctuating annually. The partly shaded forest rivers Pa’Ramudu and Pa’Masia are 7–10 m wide, shallow, slowly flowing and meandering, with stones, some cobbles and gravel, and with some submerged wood and a lot of exposed bamboo roots.

Examination of flooded wood in larger rivers at lowland reaches of Sarawak (e.g., the Sarawak River at Kuching, the Rajang River at Kapit, Batang Kayan River near Lundu) did not yield any *Ancyronyx* specimens.

##### Distribution.

*Ancyronyx procerus* was described from a tributary of the Tutoh River near Long Iman in Mulu National Park (Sarawak). The type series contains 12 specimens from the type locality and two specimens from the river Sut near Kapit (Sarawak). Recent collecting activities in northern Borneo showed that *A. procerus* is less abundant than *A. sarawacensis* or *A. acaroides*.

*Ancyronyx procerus* was recorded from Brunei, Malaysia (Pahang, Sarawak), the Philippines (Busuanga) and Vietnam (Jäch et al. 2016). The distributional data from Sabah and Terengganu represent first records from these states.

## Discussion

The species of *Ancyronyx* are usually colorful, showing various color patterns, which were originally considered to be species-specific (e.g., Jäch 1994, 2004, Kodada et al. 2014). In the present study, the use of barcoding confirmed the existence of a cryptic taxon, *A. clisteri* sp. nov., and highlighted the existence of a phenotypic plasticity regarding color patterns and other morphological characters of *A. sarawacensis* and *A. procerus* populations. Generally, in Elmidae, closely related species often replace each other along water courses; remarkably, stenothermic species of cold upper courses are usually larger, more strongly pigmented and smoother than their relatives from the lower courses (e.g., Steffan 1963, 1964; Berthélemy 1967; Jäch 1984). This obviously temperature-related phenomenon has been observed also in different populations of the same species, e.g., in *Elmis maugetii* Latreille (Knie 1977, 1978; Berthélemy 1979), *Ilamelmis foveicollis* (Grouvelle) (Jäch 1984), and it is now confirmed by molecular data for populations of *Ancyronyx sarawacensis*.

The aedeagus of *Ancyronyx* is of a trilobate type, with moderately large parameres and a short phallobase. The form of the penis in most species is simple, with sides more or less straight and narrowed apicad (e.g., *A. sarawacensis*, *A. procerus*, *A. variegatus*), but in some species (*A. acaroides* and *A. johanni* Jäch), the sides of the penis are angular and strongly produced laterad, while they are produced and rounded in *A. patrolus* Freitag & Jäch, *A. pseudopatrolus* Freitag & Jäch and *A. punktii* Freitag & Jäch. The form of the parameres is simple, and their length, setation, and shape are varying between the species.

Hayashi and Yoshitomi (2015) described a technique for endophallus extrusion, which allows a better examination of its internal morphology (bladders and sclerites) and its surface structures, which are difficult to observe when the endophallus is in resting position; in Japanese species of the genus *Zaitzeviaria* Nomura they were able to find characters useful for species identifications. The examination of an extruded endophallus in *A. sarawacensis* revealed a possible diagnostic potential in the form and position of several bladders, microsclerites and different spines. Unfortunately, it is not possible to manually evert the endophallus in all specimens. The success rate of getting the endophallus fully everted was low even when the technique described by Hayashi and Yoshitomi (2015) was used. However, these particular structures are probably of some significance also in *Ancyronyx*.

## Supplementary Material

XML Treatment for Ancyronyx clisteri

XML Treatment for Ancyronyx sarawacensis

XML Treatment for Ancyronyx procerus

## Supplementary materials

Supplementary material 1 Table S1 Data type: measurement. Explanation note: Pairwise genetic distance among specimens of *Ancyronyx* and *Graphelmis* species (Kimura 2-parameter distance) based on the 661bp barcoding fragment of the COI gene. https://binary.pensoft.net/file/380506This dataset is made available under the Open Database License (http://opendatacommons.org/licenses/odbl/1.0/). The Open Database License (ODbL) is a license agreement intended to allow users to freely share, modify, and use this Dataset while maintaining this same freedom for others, provided that the original source and author(s) are credited.Ján Kodada, Manfred A. Jäch, Hendrik Freitag, Zuzana Čiamporová-Zaťovičová, Katarína Goffová, Dávid Selnekovič, Fedor Čiampor Jr
